# Inhibition of *O*-acetylserine sulfhydrylase by fluoroalanine derivatives

**DOI:** 10.1080/14756366.2018.1504040

**Published:** 2018-09-24

**Authors:** Nina Franko, Konstantinos Grammatoglou, Barbara Campanini, Gabriele Costantino, Aigars Jirgensons, Andrea Mozzarelli

**Affiliations:** a Food and Drug Department, University of Parma, Parma, Italy;; b Latvian Institute of Organic Synthesis, Riga, Latvia;; c National Research Council, Institute of Biophysics, Pisa, Italy

**Keywords:** Fluoroalanine, cysteine biosynthesis, enzyme inhibition, pyridoxal 5′-phosphate

## Abstract

*O*-acetylserine sulfhydrylase (OASS) is the pyridoxal 5′-phosphate dependent enzyme that catalyses the formation of L-cysteine in bacteria and plants. Its inactivation is pursued as a strategy for the identification of novel antibiotics that, targeting dispensable proteins, holds a great promise for circumventing resistance development. In the present study, we have investigated the reactivity of *Salmonella enterica* serovar Typhimurium OASS-A and OASS-B isozymes with fluoroalanine derivatives. Monofluoroalanine reacts with OASS-A and OASS-B forming either a stable or a metastable α-aminoacrylate Schiff’s base, respectively, as proved by spectral changes. This finding indicates that monofluoroalanine is a substrate analogue, as previously found for other beta-halogenalanine derivatives. Trifluoroalanine caused different and time-dependent absorbance and fluorescence spectral changes for the two isozymes and is associated with irreversible inhibition. The time course of enzyme inactivation was found to be characterised by a biphasic behaviour. Partially distinct inactivation mechanisms for OASS-A and OASS-B are proposed.

## Introduction

**Scheme 1.**
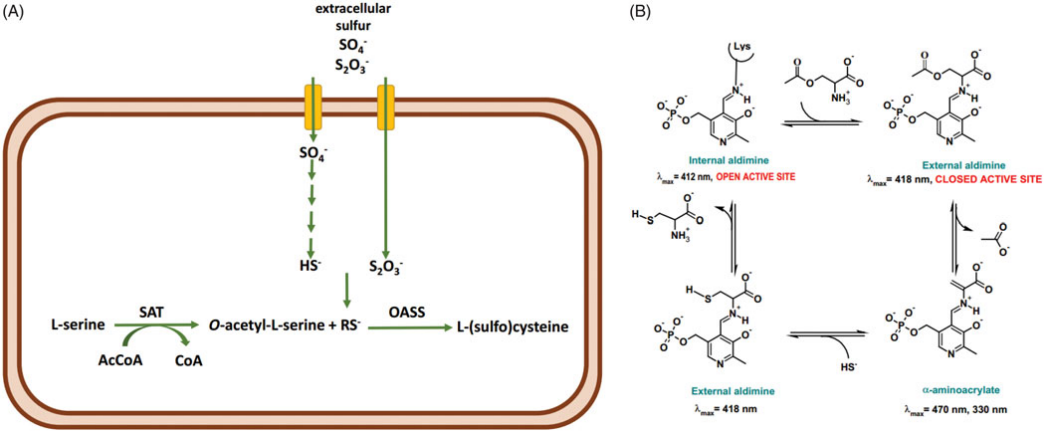
Panel A: sulfur assimilation in bacteria. Thiosulfate is an alternative substrate to bisulfide and it can only be used by the OASS-B isozyme. SAT: serine acetyltransferase; RS^-^: sulfur source, either SH^-^ or S_2_O_3_^-^. Panel B: catalytic cycle of OASS.

Sulfur is a fundamental component of many biomolecules, from amino acids to cofactors and compounds that control the redox homoeostasis. Only bacteria and plants can assimilate inorganic sulfur, either sulfate or thiosulfate, via a biosynthetic pathway leading to the formation of cysteine (Scheme 1(A))^1^.

The final step is catalysed by *O*-acetylserine sulfhydrylase (OASS), a pyridoxal 5′-phosphate (PLP)-dependent enzyme that carries out a β-replacement reaction (Scheme 1(B)). First, *O*-acetylserine forms an external aldimine that undergoes the β-elimination of an acetoxy moiety leading to the aminoacrylate Schiff’s base that is attacked by sulfide with the formation of the external aldimine of cysteine which is finally released to regenerate the internal aldimine.

OASS is present in bacteria as two isoforms, OASS-A and OASS-B, also named CysK and CysM after the coding genes. Their distribution, structural, and functional properties have been deeply investigated^2–8^. The enzyme that catalyses the synthesis of OAS, i.e. serine acetyltransferase (SAT), is able to form a high-affinity complex with OASS-A but not with OASS-B. Because SAT binding to OASS-A involves anchoring of its C-terminus in OASS-A active site, complex formation leads to the competitive inhibition of OASS-A activity. Both isoforms are present in bacteria but not in humans. Bacteria knocked-out for *cysK* and *cysM* exhibit a phenotype with reduced virulence, compromised fitness, and decreased antibiotic resistance^9^^,^^10^. For these reasons, OASS has been the target of multiple medicinal chemistry efforts to identify reversible inhibitors potentially useful as antibiotics or enhancers of antibiotic activity^11^^,^^12^. Initially, pentapeptides that mimic the inhibitory interaction between OASS-A and the C-terminus of SAT were developed^13^^,^^14^. Then, more potent and isoform-specific peptidomimetic compounds based on cyclopropane derivatives were generated, with some of them exhibiting nanomolar K_i_ against OASS-A from *S.* Typhimurium^12^^,^^15^.

The debate between pros and cons of reversible and irreversible enzyme inhibitors has been developing along the history of medicinal chemistry with alternative views^16^^,^^17^. Reversible inhibitors either directed to the active site or allosteric sites are thought to be more specific, thus less prone to toxicity effects due to unwanted off-target reactions, which are typical of irreversible inhibitors that exploit the intrinsic reactivity of protein residues, such as cysteines or cofactors. However, the concentration of irreversible inhibitors needed to inactivate enzymes is usually lower than the concentration required by reversible inhibitors and this can potentially lead to an increased therapeutic index. Two classes of irreversible inhibitors have been developed: mechanism-based inactivators and affinity labels^18^^,^^19^.

**Scheme 2.**
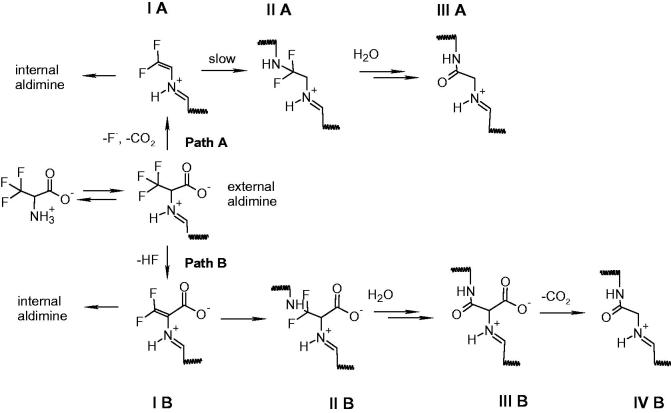
Possible inactivation pathways of PLP-dependent enzymes by *β*,*β*,*β*-trifluoroalanine.

Monohalogenated, dihalogenenated and trihalogenated (either chloro-based or fluoro-based) alanines have been exploited as mechanism-based inhibitors of pyridoxal 5′-phosphate (PLP)-dependent enzymes, including γ-cystathionase^20^^,^^21^, alanine racemase^22^, tryptophan synthase and tryptophanase^23^^,^^24^, 8-amino 7-oxonononatote synthase^25^, ornithine decarboxylase^26^, aspartate aminotransferase^27^, and kynurenine transaminase^28^. The reaction mechanism and the extent of inactivation are different depending on the number of halogen substituents^29^^,^^30^ and enzyme^25^. A widely accepted mechanism of inhibition by halogenated alanines includes the formation of an external aldimine with PLP (transaldimination), followed by alpha-proton abstraction and elimination of either HF or HCl with the formation of an unsaturated Schiff’s base, i.e. α-aminoacrylate-PLP complex (Scheme 2, path B). Another possible mechanism of inactivation is fluorodecarboxylation mechanism, leading to an unsaturated Schiff’s base (Scheme 2, path A)^25^^,^^31^. In both types of inhibition, the unsaturated Schiff’s base is the key partitioning intermediate and the following steps are strongly dependent on the active site environment and on the halogenated alanine, usually involving an attack of the active site lysine on the double bond, followed by chemical rearrangements that cause the release of further halogen ions and formation of stable derivatives that inactivate the enzyme.

In the present study, we investigated the reactivity of mono and trifluoroalanine derivatives with OASS-A and OASS-B in order to identify suitable mechanism-based irreversible inhibitors.

## Material and methods

### Reagents

If not otherwise specified, chemicals were purchased from Sigma-Aldrich (St. Louis, MO, USA) at the highest available quality. Ninhydrin was purchased from Apollo Scientific (Stockport, UK).

### Protein preparation

**Figure 1.**
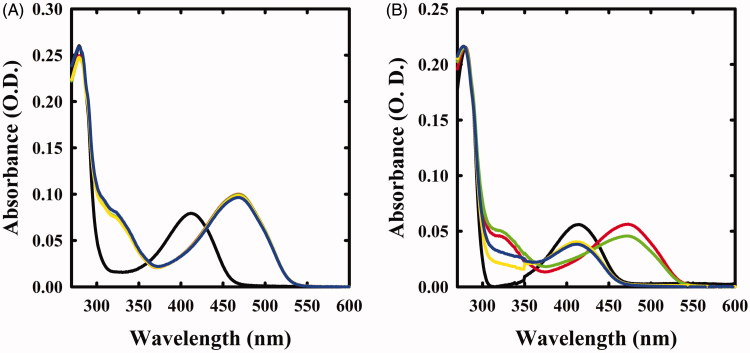
Spectral changes of OASS in the presence of F-Ala. Panel A: OASS-A in the absence of reagent (black line), 1 min (red line), 1 h (green line), 5 h (yellow line), and 21 h (blue line) after addition of 1 mM F-Ala. Panel B: OASS-B in the absence of reagent (black line), 1 min (red line), 1 h (green line), 3 h (yellow line), and 7 h (blue line) after addition of 1 mM F-Ala.

Recombinant *ST*OASS-A and *ST*OASS-B were expressed in *Escherichia coli* BL21(DE3) and purified as described previously^32^. Briefly, His-tagged proteins were purified using ion metal affinity chromatography on immobilised Co^2+^ ions (Talon Technology, Clontech Laboratories, Inc., Mountain View, CA, USA). His-tag was removed at 37 °C by factor Xa in a 1:200 ratio with protein in 20 mM HEPES, 100 mM NaCl, and 4 mM CaCl_2_, pH 7.5. Proteins were more than 93% pure accordingly to SDS-PAGE (see Figure 1SM). Protein concentration was determined by extinction coefficients of the bound PLP, that are 9040 M^−1 ^cm^−1^ at 412 nm for OASS-A and 6800 M^−1 ^cm^−1^ at 414 nm for OASS-B. OASS-A was stored in 10 mM HEPES, pH 8.0 and OASS-B in 5 mM HEPES, pH 8.0.

### Monitoring the reactions by absorbance spectroscopy

Absorbance spectra were recorded with a Cary4000 spectrophotometer (Agilent Technologies) on solutions containing 5–10 µM enzyme, 1 mM fluoroalanine derivative, 100 mM HEPES, pH 7.0, at room temperature. Spectra were corrected for buffer contribution. The time course of band disappearance at a fixed wavelength was fitted to a single exponential decay equation:

(1) $$A_{t}=A_{0}+{A_{i}}^{-k_{obs}\;\cdot \;t},$$

where *A_t_* is absorbance at time *t*, *A*_0_ is the absorbance at infinite time, *A_i_* is the total absorbance change and *k_obs_* is the rate constant**.**

### Monitoring the reactions by fluorescence spectroscopy

Fluorescence emission spectra were recorded with a FluoroMax-3 fluorimeter (HORIBA) on solutions containing 1 µM enzyme, 1 mM fluoroalanine, 100 mM HEPES, pH 7.0, at room temperature, upon excitation at 412 nm, with slit_ex_ = slit_em_ = 6 nm. Spectra were corrected for buffer and compound contribution.

### Activity assays

Enzyme activity under steady-state conditions was measured by a discontinuous method, following the quantification of cysteine concentration by the method developed by Gaitonde^33^, adjusted to 96-well plate format. Briefly, reaction was initiated by addition of 0.6 mM Na_2_S to a solution containing 6 nM OASS-A/OASS-B, 1 mM OAS, 100 mM HEPES, pH 7.0, a 10-fold excess of BSA with respect to enzyme concentration to prevent enzyme adhesion to well wall and variable concentrations of potential inhibitors. Aliquots (60 µL) were withdrawn at time intervals and the reaction was stopped in PCR tubes containing 60 µL of acetic acid. 60 µL of ninhydrin were added by a multichannel pipette and the mixture was heated at 100 °C for 10 min in a thermal cycler. The solutions were cooled down on the ice and 46 µL were added to the wells of a 96-well plate containing 154 µL of cold ethanol. The absorbance at 550 nm was measured using a plate reader (Halo LED 96, Dynamica Scientific, Newport Pagnell, UK). Time courses were collected at least in duplicate. The amount of cysteine produced at each time point was calculated from a calibration curve, and data were fitted to a linear equation to calculate the initial rate of cysteine production. The fractional velocity was determined as a function of inhibitor concentration and IC_50_ was calculated using Equation (2):

(2) $$\frac{v_{i}}{v_{0}}=\frac{1}{1+(\frac{\left[I\right]}{IC50})},$$

where *v*_0_ is the initial rate in the absence of inhibitor and *v*_i_ is the initial rate in the presence of inhibitor at concentration [*I*].

### Inactivation kinetics

OASS (45 µM) was incubated with various concentrations of β,β,β-trifluoroalanine in 400 mM HEPES, pH 7.0, at room temperature. Enzyme activity at different time points was assayed as described above upon a 5000-fold dilution with a solution containing 100 mM HEPES, 90 nM BSA, pH 7.0. Inactivation kinetics were collected at different concentrations of β,β,β-trifluoroalanine, and k_obs_ values were determined by fitting data to the equation of a single exponential decay:

(3) $$\frac{v_{i}}{v_{0}}={A+B\cdot e}^{-k_{obs}\;\cdot \;t},$$

where *v*_0_ is the initial rate in the absence of inhibitor, *v*_i_ is the initial rate in the presence of inhibitor at concentration [*I*], *A* is an off-set, *B* is the amplitude, and *k_obs_* is the rate constant.

Enzyme activity was also measured upon 85 h of incubation in the presence of β,β,β-trifluoroalanine, removal of ligand by ultrafiltration, in the absence and presence of 1 µM PLP added to the activity assay mixture. The fractional activity was calculated with respect to enzyme incubated under the same conditions in the absence of β,β,β-trifluoroalanine.

Potency of inhibitor was determined as the ratio *k_inact_*/*K_I_* that for OASS-A was determined from Equation (4):

(4) $$k_{obs}=\frac{k_{\text{inact}}\times [I]}{K_{I}+[I]},$$

where *k_obs_* is the observed rate constant of inactivation at inhibitor concentration [I], *k_inact_* is inactivation rate constant and *K_I_* is concentration of inhibitor that yields *k_obs_* = ½ *k_inact_*. *k_inact_*/*K_I_* for OASS-B was determined using Equation (5):

(5) $$\frac{k_{obs}}{\left[I\right]}=\frac{k_{\text{inact}}}{K_{I}}.$$

## Results and discussion

### Monitoring the reaction of monofluoro- and trifluoroalanine with OASS-A and OASS-B by absorbance and fluorescence spectroscopy

The absorption spectra of OASS-A and OASS-B exhibit a band at 412 nm, attributed to the ketoenamine tautomer of the internal aldimine (Figure 1). When 1 mM monofluoroalanine (F-Ala) was added, the band shifted to 470 nm, a band attributed to the α-aminoacrylate Schiff’s base (Figure 1(A))^5^^,^^6^^,^^34^. This species, that was stable for at least 21 h, is generated from the β-elimination reaction of HF, similar to the β-elimination reaction observed for the acetoxy moiety of the natural substrate OAS. When the same reaction was carried out on OASS-B, a rapid formation of the α-aminoacrylate was observed, followed by the slow reappearance of the band at 412 nm (Figure 1(B)) and a broad absorbance between 300 and 350 nm, where pyruvate absorbs. This finding indicates that the α-aminoacrylate Schiff’s base of OASS-B decomposes to pyruvate and ammonia much faster than the α-aminoacrylate Schiff’s base of OASS-A. The same behaviour was observed in the formation of aminoacrylate with *O*-acetylserine^3^.

**Figure 2.**
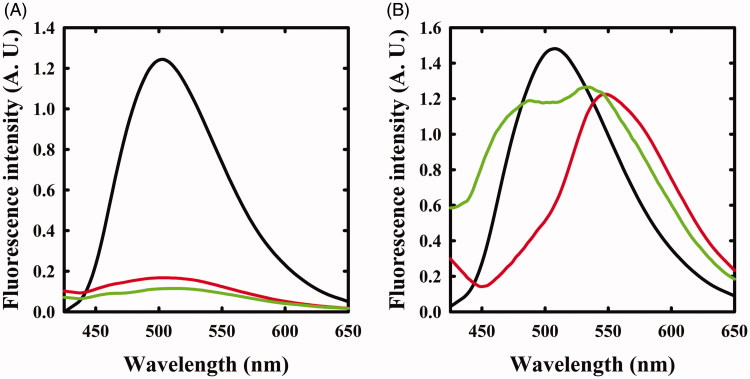
Fluorescence emission spectra of OASS upon excitation at 412 nm in the absence and presence of F-Ala. Panel A: OASS-A in the absence of reagent (black line), 1 min (red line), and 4 h (green line) after addition of 1 mM F-Ala. Panel B: OASS-B in the absence of reagent (black line), 1 min (red line), and 3 h (green line) after addition of 1 mM F-Ala.

The same reaction was also monitored by fluorescence spectroscopy. The emission spectrum of OASS-A, upon excitation at 412 nm, was centred at 505 nm^8^^,^^35^ and disappeared upon addition of F-Ala (Figure 2(A)), in agreement with the previous studies indicating that the fluorescence quantum yield of the α-aminoacrylate is much lower than that of the internal aldimine^8^. When the reaction with F-Ala was carried out on OASS-B (Figure 2(B)), only a shift to 550 nm of the emission peak was observed, in agreement with our previous work^7^. This emission was attributed to an α-aminoacrylate located in an active site with a different microenvironment compared to OASS-A isozyme.

Given the observed spectral changes, it can be concluded that F-Ala behaves as a substrate analog of OASS, as previously observed for β-chloroalanine^30^ and that the intermediate α-aminoacrylate is oriented within the active site in such a way to disfavour the reaction with any active site residue. This finding is not surprising considering that OASS has evolved to stabilise an α-aminoacrylate intermediate ready to react with the incoming nucleophilic sulfide. In addition, it has been reported for alanine racemase^29^^,^^36^ that the partition ratio between α-aminoacrylate hydrolysis and Michael addition on the adduct formed from the F-Ala is 820:1, a further indication of the very poor reactivity of this species in the enzyme active site.

**Figure 3.**
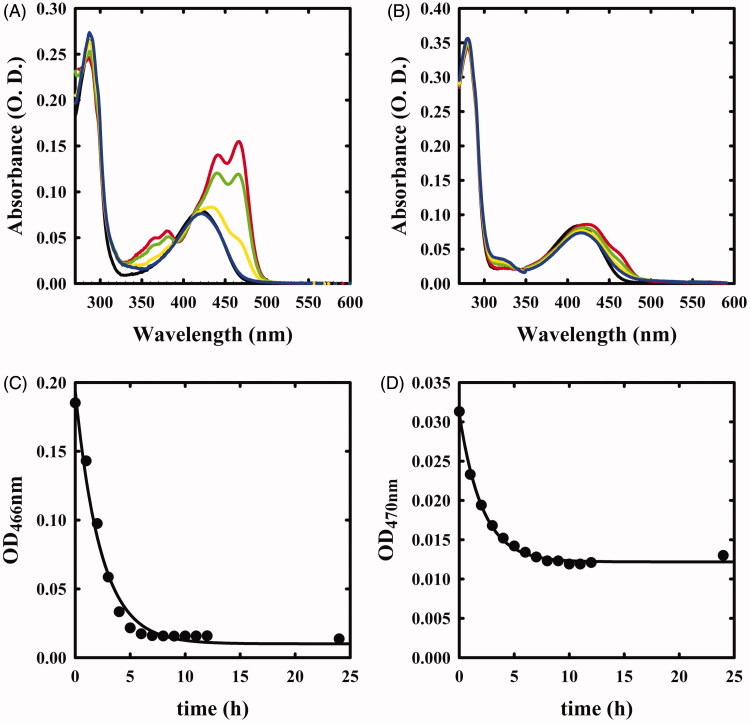
Absorbance spectra of OASS in the absence and presence of 1 mM triF-Ala. Panel A: absorbance spectrum of OASS-A in the absence of reagent (black line), 1 min (red line), 1 h (green line), 3 h (yellow line), and 7 h (blue line) after addition of the reagent. Panel B: absorbance spectrum of OASS-B in the absence of reagent (black line), 1 min (red line), 1 h (green line), 3 h (yellow line), and 7 h (blue line) after addition of the reagent. Panel C: time course of spectral changes of OASS-A, monitored at 466 nm. Panel D: time course of spectral changes of OASS-B, monitored at 457 nm starting 1 min after the addition of triF-Ala. Data were fitted to a monoexponential decay.

We then investigated the reactivity of OASS-A and OASS-B with 1 mM β,β,β-trifluoroalanine (triF-Ala), a well-known suicide substrate of PLP-dependent enzymes^20^^,^^23–26^^,^^29^^,^^30^. The absorbance spectra collected as a function of time exhibited a complex behaviour (Figure 3). Immediately upon the addition of the reagent to OASS-A, two prominent peaks appeared, centred at 440 and 466 nm, and minor bands at 360 and 380 nm, as already observed for the reaction of triF-Ala with alanine racemase^30^, indicative of a species with extended conjugation. Absorbance intensity was found to be nearly independent of reagent concentration between 1 and 10 mM (data not shown), suggesting that all enzyme sites have reacted with the reagent forming a metastable species. The absorbance intensity at 466 nm slowly decreased with the *k_obs_* of 0.43 h^−1^, with formation of a band centred at 412 nm (Figure 3(C)).

The reaction of OASS-B with triF-Ala was accompanied by much smaller spectral changes in the range 400–500 nm (Figure 3(B)), independent of a ten-fold increase in triF-Ala concentration (data not shown). The time course for intermediate decay at 457 nm was similar to OASS-A (*k_obs_* 0.48 h^−1^) and was accompanied by the increase in the absorbance in the range 300–350 nm, suggesting the production of a keto-acid, possibly difluoropyruvate.

**Figure 4.**
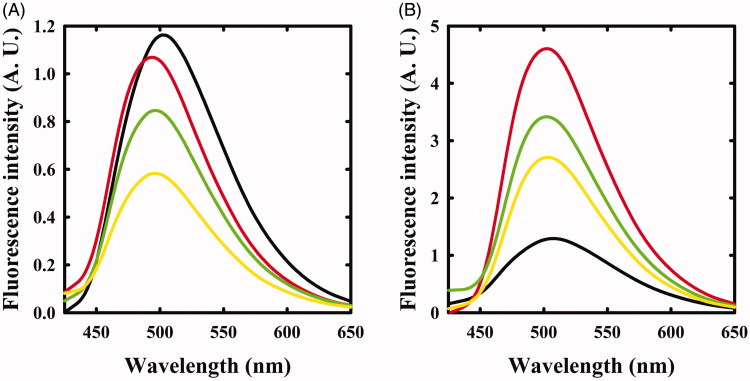
Fluorescence emission spectrum of OASS in the absence and presence of 1 mM triF-Ala. Emission spectra were recorded upon excitation at 412 nm. Panel A: OASS-A in the absence of reagent (black line), 1 min (red line), 4 h (green line), and 6 h (yellow line) after addition of the reagent. Panel B: OASS-B in the absence of reagent (black line), 1 min (red line), 3 h (green line), and 7 h (yellow line) after addition of the reagent.

The reaction of triF-Ala with OASS-A was also monitored by fluorescence emission of the cofactor (Figure 4(A)). Immediately upon reaction, the emission band measured upon excitation at 412 nm was blue shifted to 495 nm with only a small decrease in emission intensity. This finding confirms that the species that is formed immediately upon exposure to triF-Ala is not the α-aminoacrylate Schiff’s base, in agreement with absorbance data. The emission band slowly decreased as a function of time with a kinetics that parallels that observed for the disappearance of the absorption bands at 440 and 466 nm. However, after 6 h incubation, the initial emission spectrum is not recovered, differently from what was observed by absorbance spectroscopy. This suggests that the species that forms and absorbs at 412 nm is not the internal aldimine.

When the reaction of triF-Ala with OASS-B was monitored by recording emission spectra upon excitation at 412 nm (Figure 4(B)), a different behaviour with respect to OASS-A was observed. The intensity of emission band immediately increases, then slowly decreases. Changes in intensity are accompanied by a small blue shift to 501 nm that shifts back slowly to 505 nm after 7 h of incubation. The intense emitting species might be an external aldimine, because in most PLP-dependent enzymes, including OASS, the external aldimine is endowed by high fluorescence intensity^7^^,^^8^. The small blue shift suggests the formation of a transient species.

### Monitoring the reaction of F-Ala and triF-Ala with OASS-A and OASS-B by activity assays

**Figure 5.**
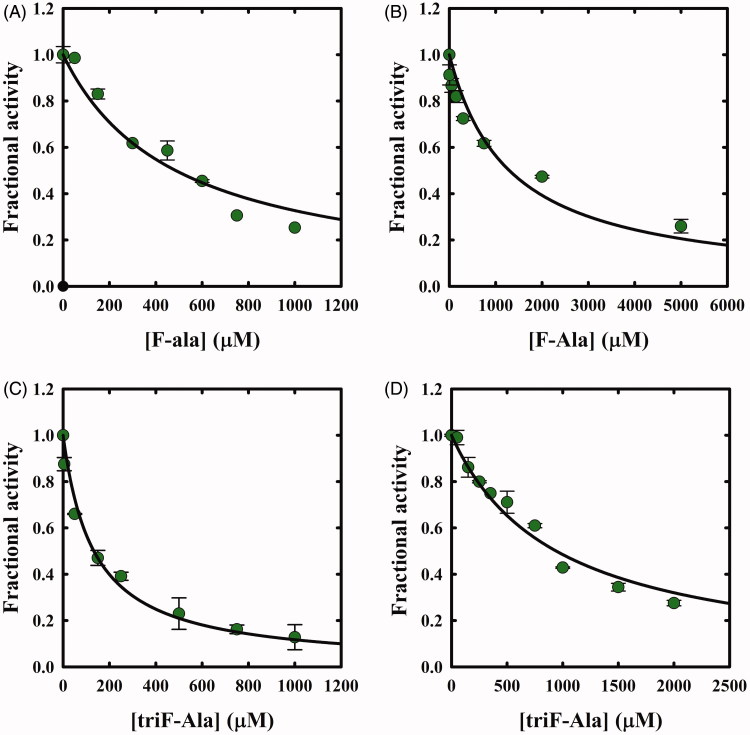
Dependence of OASS fractional activity on the concentration of fluoroalanine derivatives. (A) OASS-A and F-Ala; (B) OASS-B and F-Ala; (C) OASS-A and triF-Ala; (D) OASS-B and triF-Ala. The IC_50_ was obtained by fitting data points to Equation (2). The calculated IC_50_ values for F-Ala were 480 ± 50 µM and 1290 ± 230 µM for OASS-A and OASS-B, respectively, and for triF-Ala 130 ± 10 µM and 940 ± 60 µM, for OASS-A and OASS-B, respectively.

The potential inhibitory action of F-Ala and triF-Ala on OASS-A and OASS-B was investigated by carrying out two distinct experiments. First, OASS-A and OASS-B were assayed within a few seconds from the exposure to increasing concentrations of F-Ala. IC_50_ values of 480 ± 50 µM and 1290 ± 230 µM were determined, respectively (Figure 5(A,B)). The same experiment was carried out for triF-Ala determining IC_50_ values of 130 ± 10 µM and 940 ± 60 µM, for OASS-A and OASS-B, respectively (Figure 5(C,D)).

**Figure 6.**
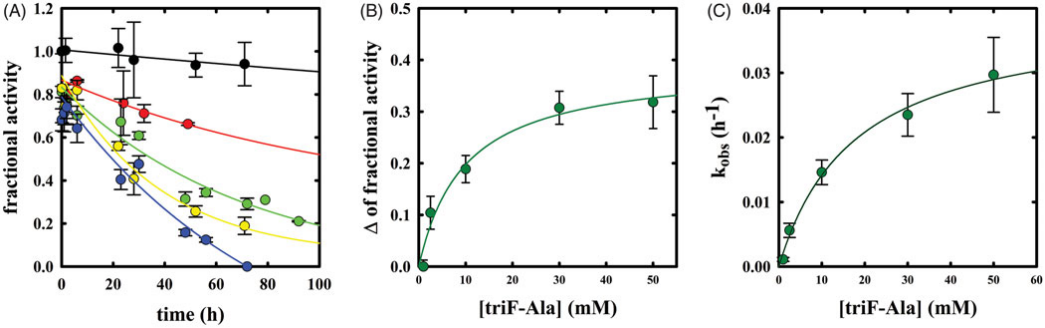
Panel A: Inactivation kinetics of OASS-A upon prolonged incubation time with 1 (black), 2.5 (red), 10 (green), 30 (yellow) and 50 mM (blue) triF-Ala. Panel B: Extent of OASS-A inactivation determined upon completion of the fast phase. Panel C: dependence of k_obs_ on the concentration of triF-Ala.

Whereas F-Ala inhibits OASS by competing with the substrate and is slowly degraded, as reported above and observed for other PLP-dependent enzymes, triF-Ala might inhibit OASS by irreversible inactivation. To detect whether this is the case, we monitored the kinetics of OASS-A inactivation in the presence of 1, 2.5, 10, 30, and 50 mM triF-Ala (Figure 6). At each time point, the enzyme activity was determined upon a 5000-fold dilution of the reaction mixture. The time course of enzyme inhibition was found to be clearly biphasic. The rate constant of the very fast phase cannot be measured, whereas its amplitude was found to be dependent on reagent concentration (Figure 6(B)) and approaching saturation. The rate of the slow phase depends hyperbolically on reagent concentration, as expected for suicide substrates. The second-order rate constant *k_inact_*/*K_I_* was determined to be 3.95 × 10^−5 ^min^−1 ^mM^−1^, indicating poor effectiveness in enzyme inactivation.

When the time dependence of spectral changes of OASS-A (Figure 3(A,C)) is compared with that of enzyme inactivation (Figure 6(A)), at least three phases in the reaction of triF-Ala with the enzyme are identified: (i) a fast phase completed in a few seconds in which a species is built-up absorbing at 440–470 nm; (ii) an intermediate phase completed in about 10 h where the species absorbing at 440–470 disappears with the concomitant appearance of a species at 412 and is accompanied by minor decrease in enzyme activity, and (iii) a very slow phase where most of enzyme inactivation takes place. It is worth stressing that the very fast phase of enzyme inhibition monitored by changes in the absorption spectrum (Figure 3(A)) is completely reversible within 8 h for inhibitor concentrations up to 1 mM (Figure 6(A)), thus the IC_50_s (Figure 5) are a good estimate of the relative affinity of F-Ala and triF-Ala for the enzyme. The different phases observed monitoring the inactivation kinetics and absorbance spectra likely arise from the different ratio between enzyme concentration (10 µM in the absorbance spectra and 45 µM in the activity assays) and inactivator concentration.

We verified that the inhibition is irreversible by assaying the enzyme after 85-h reaction with 10 mM triF-Ala, complete removal of ligand by extensive ultrafiltration and incubation in the presence of saturating PLP concentration. We found that the remaining enzyme activity (about 25%) did not change upon ultrafiltration and incubation with PLP. This result indicates that enzyme inactivation brought about by triF-Ala does not involve displacement of the cofactor from the active site but is rather caused by an irreversible covalent modification of active site residue(s).

**Figure 7.**
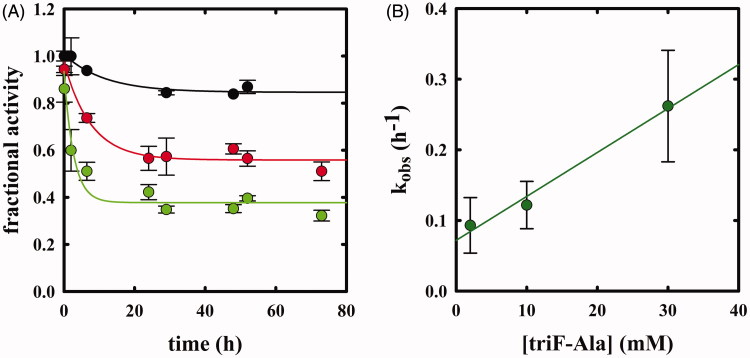
Panel A: Inactivation kinetics of OASS-B upon prolonged incubation time with 2 (black), 10 (red) and 30 mM (green) triF-Ala. Panel B: dependence of k_obs_ to the concentration of triF-Ala.

The kinetics of enzyme inactivation by triF-Ala was also measured on OASS-B (Figure 7(A)), observing again a biphasic enzyme inactivation. The amplitude of the fast phase is smaller but still dependent on reagent concentration, and the rate of the slow phase is about 10-fold faster than that observed for OASS-A and dependent on reagent concentration (Figure 7(B)). The dependence between *k_obs_* and [*I*] appears to be linear, likely because the saturation conditions were not reached. *k_inact_*/*K_I_* was therefore estimated using the Equation (5) that describes single step inactivation and is 10.3 × 10^−5 ^min^−1 ^mM^−1^.

In the case of OASS-B, there are two phases in the reaction of triF-Ala with OASS-B: a fast phase that occurs immediately upon addition of the reagent that can be followed by absorbance changes at 470 nm, followed by the slow disappearance of the shoulder at 470 nm that parallels the enzyme inactivation. Differently, from the OASS-A isozyme, the disappearance of the shoulder at 470 nm is accompanied not only by the recovery of the peak at 412 nm but also by the formation of a peak at about 320 nm, likely due to the accumulation of difluoropyruvate.

Several fluoroalanine derivatives were tested (Table 1) to assess the effect of chain extension branching and fluorine substitution (compounds **1–4**). None of these modifications resulted in an improvement in inactivation for both isozymes. In a further effort, series of trifluoroalanine derivatives where the carboxylic moiety was substituted with bioisosters were tested (Table 2). These analogues were aimed to identify a scaffold with options to tune activity and selectivity of the inhibitor which is not possible for trifluoroalanine. A set of amides with different *N-*substitution patterns **5–11** was prepared. Again, negligible effects on enzyme activity were observed, with the exception of hydroxamic acid **10** that caused small absorbance changes in OASS-A (Figure 2SM) and about 14% decrease in enzyme activity.

**Table 1.** Reactivity between OASS and alanine derivatives.


			Reactivity against OASS-A		Reactivity against OASS-B	
	R_1_	R_2_	% Inhibition*	Spectral changes	% Inhibition*	Spectral changes
**1**	CF_3_CH_2_	H	N.S.	No	18 ± 2	No
**2**	CF_3_	Me	N.S.	No	N.S.	No
**3**	CHF_2_CH_2_	H	12 ± 1	No	16 ± 5	No
**4**	EtO_2_CCF_2_**	H	14 ± 1	No	N.S.	No

* % Inhibition was evaluated after 6 h incubation of the enzyme with 1 mM inhibitor, following 5000-times dilution for the assay (0.2 μM inhibitor in the assay) in two replicates. Percent inhibition of 10% or lower was considered not significant (N.S.).

** As TFA salt.

**Table 2.** Reactivity between OASS and β,β,β-trifluoroalanine carboxylate bioisosters.


		Reactivity against OASS-A		Reactivity against OASS-B	
	R	% Inhibition*	Spectral changes	% Inhibition*	Spectral changes
**5**	–C(=O)NHMe	N.S.	No	N.S.	No
**6**	–C(=O)NMe_2_	N.S.	No	N.S.	No
**7**	–C(=O)NHPh	N.S.	No	N.S.	No
**8**	–C(=O)NBn_2_	10 ± 2	No	N.S.	No
**9**	–C(=O)NH(*p*-_6_H_13_-Ph)	N.S.	No	12 ± 7	No
**10**	–C(=O)NHOH	15 ± 5	Yes	N.S.	No
**11**	–C(=O)NH_2_	N.S.	No	N.S.	No
**12**	–P(=O)(OH)_2_	N.S.	No	N.S.	No

* % Inhibition was evaluated after 6 h incubation of the enzyme with 1 mM inhibitor, following 5000-times dilution for the assay (0.2 μM inhibitor in the assay) in two replicates. Percent inhibition of 10% or lower was considered not significant (N.S.).

On the basis of the spectral and kinetic data obtained by monitoring the reactivity of triF-Ala with OASS and taking into account reaction schemes previously proposed in the reaction of triF-Ala with other PLP-dependent enzymes^20^^,^^22^^,^^24^^,^^25^^,^^29^^,^^30^^,^^36^, we propose an inactivation mechanism for OASS-A that is similar to the mechanism proposed for alanine racemase^30^ (Scheme 2, path B). It is unlikely that the inactivation follows path A on Scheme 2 because decarboxylation does not occur in the catalytic cycle of OASS.

After the formation of the external aldimine, the elimination of HF leads to the formation of β,β-difluoro-α,β-unsaturated imine (intermediate **IB,**Scheme 2). The intermediate **IB** contains delocalised electrons that might account for the spectrum with bands at 440 and 466 nm. This species can be hydrolysed, with the release of difluoropyruvate and ammonia, leading to an internal aldimine. The ratio between hydrolysis and nucleophilic attack, which both lead to the decrease in the absorbance at 450–470 nm, is dictated by the concentration of inhibitor, geometry of active site residues and water accessibility. In the case of covalent modification, the species undergoes the Michael attack by the enzyme nucleophile, likely the active site lysine, leading to intermediate **IIB**. Formation of an intermediate absorbing at about 410–420 nm but distinct from the internal aldimine is supported by the fluorescence emission spectra (Figure 4(A)) where a decrease in the emission of a species at 495 nm was measured. Based on the literature data^25^, we propose that this species is unstable and undergoes the addition of water on β-carbon, followed by the elimination of two fluoride ions, forming the intermediate **III B**. This step has been observed on alanine racemase after partial denaturation with sodium borohydride, suggesting that the active site needs to be open and accessible to water. The slow fluoride elimination is controlled by a conformational change occurring in the active site that limits water accessibility. The final step might be the loss of the carboxylic moiety with formation of intermediate **IV B.**

Inactivation of OASS-B by triF-Ala follows the reaction scheme of the OASS-A isozyme although with different interconversion rates of intermediates. Indeed, in this case, external aldimine is the predominant species, as observed in the absorbance and fluorescence emission spectra. Elimination of HF leads to the formation of β,β-difluoro-α,β-unsaturated imine (**I B**) that does not accumulate to any significant extent. For this reason, the fraction of inactivated enzyme at this stage is small, differently from what observed for OASS-A isozyme where more intermediate accumulates. The Michael attack by the catalytic lysine leads to the elimination of difluoropyruvate, which absorbs at 320 nm, and ammonia. This reaction is more efficient for the OASS-B isozyme with respect to the OASS-A isozyme, thus preventing accumulation of the I B intermediate. Inactivation takes place by the same mechanism proposed for OASS-A by an attack of an active site nucleophile, likely the catalytic lysine, on the β-carbon.

## Conclusions

The search for reversible and irreversible inhibitors of OASS-A and OASS-B is dictated by the relevance of these enzymes in the biosynthesis of cysteine in bacteria and by their absence in mammals. Cysteine depletion is associated with a decrease in bacterial fitness, thus enhancing antibiotics efficacy. Whereas reversible inhibitors for OASS-A and OASS-B with nanomolar/micromolar affinities have been identified^11^^,^^12^^,^^37^, studies aiming at developing irreversible inhibitors are still lacking. The present investigation was aimed to fill this gap by exploring the reactivity of a class of compounds, fluoroalanine derivatives that are well-known inhibitors of PLP-dependent enzymes. We found that monofluoralanine is a weak substrate analogue for both isozymes, whereas trifluoroalanine acts as irreversible, although inefficient, inhibitor.

## Supplementary Material

Supplemental Material
