# Supplementary material for: Inhibition of O-acetylserine sulfhydrylase by fluoroalanine derivatives
Source: J Enzyme Inhib Med Chem. 2018 Sep 24;33(1):1343–51. doi: 10.1080/14756366.2018.1504040 (PMC6161599; doi:10.1080/14756366.2018.1504040)

-Inhibition of *O*-acetylserine sulfhydrylase by fluoroalanine derivatives – supplemental material

Nina Franko^[#], Konstantinos Grammatoglou°[#], Barbara Campanini^*,

Gabriele Costantino^, Aigars Jirgensons° and Andrea Mozzarelli^ς

^*Department of Food and Drug, University of Parma, Parma Italy,*

°*Latvian Institute of Organic Synthesis, Riga, Latvia,*

ς*National Research Council, Institute of Biophysics, Pisa, Italy*

[#] The authors contributed equally to the work

Corresponding author: Barbara Campanini, Dipartimento di Scienze degli Alimenti e del Farmaco, Università di Parma, Parco Area delle Scienze 23/A, Parma Italy, Email: barbara.campanini@unipr.it

Purity of OASS on SDS PAGE

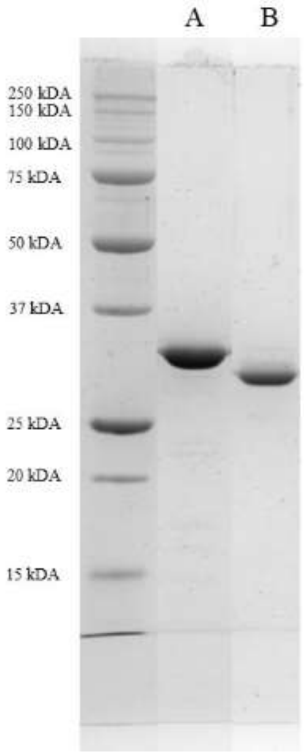

Figure 1SM. SDS-PAGE of OASS-A and OASS-B. Enzyme purity was evaluated to be more that 93%.

Reactivity between OASS-A and compound 10

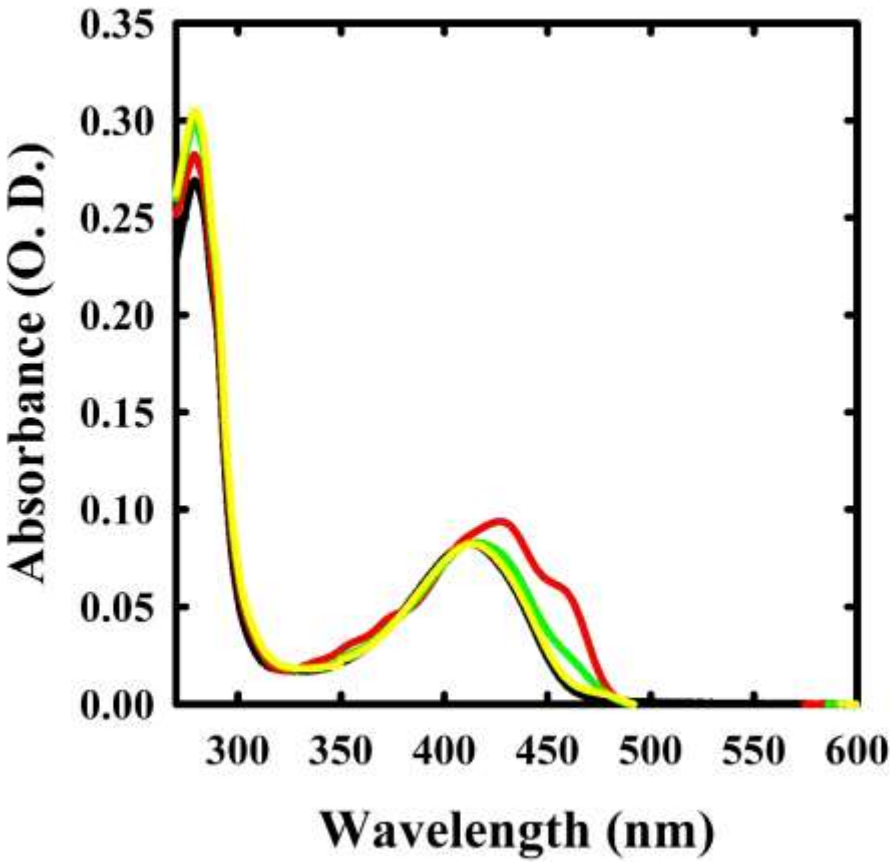

Figure 2SM. Spectral changes for the reaction between OASS-A and 1 mM compound **10**. OASS-A in the absence of reagent (black line) and 1 min (red line), 30 min (green line) and 1 h (yellow line) after compound 10 addition.

Synthesis of alanine derivatives

General Information: Commercially available reagents were used without further purification, 2-amino-4,4,4-trifluorobutanoic acid (1), 2-amino-3,3,3-trifluoro-2-methylpropanoic acid (2), 2-amino-4,4-difluorobutanoic acid (3) and (1-amino-2,2,2-trifluoroethyl)phosphonic acid (12) were purchased from Enamine Ltd. All air or moisture-sensitive reactions were carried out under an argon atmosphere using oven-dried glassware. Flash chromatography was carried out using Merck Kieselgel (230–400 mesh). Thin layer chromatography was performed on silica gel and was visualized by staining with KMnO4. NMR spectra were recorded on a Varian Mercury spectrometer (400 MHz) and a Bruker Fourier spectrometer (300 MHz) with chemical shift values (δ) in ppm relative to TMS using the residual chloroform signal as an internal standard. HRMS were obtained using a Q-TOF micro high resolution mass spectrometer with ESI (ESI+/ ESI-).

Boc protection of 3,3,3-trifluoro alanine: To a solution of amino acid (1 equiv.) in 1:1 mixture of THF/H_2O (0.27M), $NaHCO_3$ (3 equiv.) and Boc_2O (1.4 equiv.) were added consecutively at 0°C. After 30 min, the solution was stirred overnight at room temperature. The turbid solution was extracted with Et_2O (x2). The aqueous layer was acidified to pH = 4 by careful addition of $KHSO_4$ at 0°C and then extracted with DCM (x3). The combined organic phase was dried and evaporated under reduced pressure to give N-Boc-(3,3,3-trifluoro alanine) as white solid.

^{1}H NMR (400 MHz, DMSO-d_6) δ 8.00 (d, J = 9.4 Hz, 1H, N<u>H</u>-Boc), 4.87 (q, J = 8.8 Hz, 1H, -C<u>H</u>-), 1.37 (s, 9H, -(C<u>H</u>$_3$)$_3$) (-COO<u>H</u> not visible due to deuterium exchange). ^{13}C NMR (101 MHz, DMSO-d_6) δ 166.3, 155.6, 122.3, 79.8, 55.1, 28.4.

tert-butyl (1,1,1-trifluoro-3-(hydroxyamino)-3-oxopropan-2-yl) carbamate: CDI (1.5 equiv.) was added to a solution of N-Boc-(3,3,3-trifluoro alanine) (1 equiv.) in dry tetrahydrofuran (THF). The reaction mixture was stirred for 1 h. Powdered hydroxylamine hydrochloride (2 equiv.) was added. The resulting mixture was stirred overnight (ca. 16 h). The mixture was diluted with 5% aq. $KHSO_4$ and extracted with EtOAc (x2). The combined organic phase was washed with brine and dried over Na_2SO_4. The extract was filtered and concentrated in vacuo to give the product hydroxylamine.

^{1}H NMR (400 MHz, DMSO-d6) δ 11.10 (s, 1H, -OH), 9.30 (d, J = 1.1 Hz, 1H, -NH-OH), 7.80 (d, J = 10.0 Hz, 1H, -NH-Boc), 4.75 (t, J = 8.6 Hz, 1H, -CH-), 1.36 (s, 9H, -$(CH_3)_3$). ^{13}C NMR (101 MHz, DMSO-d_6) δ 160.4, 155.2, 125.2, 79.8, 53.3, 28.3.

2-amino-3,3,3-trifluoro-N-hydroxypropanamide (10): To a solution of tert-butyl (1,1,1-trifluoro-3-(hydroxyamino)-3-oxopropan-2-yl) carbamate in dry DCM, excess TFA (>100 equiv.) was added and stirred for 1 h at r.t.. Then excess reagent and solvent were removed under vacuum. The residue was dissolved in DCM and evaporated (x3) to obtain the product as a TFA salt.

^{1}H NMR (400 MHz, DMSO-*d*6) δ 11.73 (s, 1H, -OH), 9.09 (t, *J* = 1.3 Hz, 1H, -NH-OH), 5.18 (d, *J* = 8.1 Hz, 1H, -CH-), 4.72 (d, *J* = 8.1 Hz, 2H, -NH_2). ^{13}C NMR (101 MHz, DMSO-d6) δ 170.7, 119.8, 60.0.

HR-MS (ESI-TOF) m/z: Calcd for $C_3H_4N_2O_2F_3$ 157.0225; Found $[M-H]^-$ 157.0227

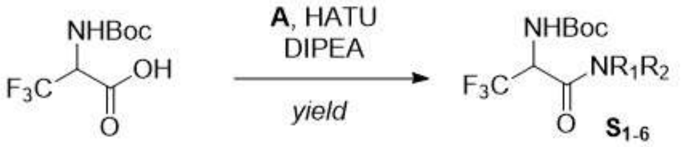

		yield
**A:** NH_4Cl	**S_1** : R_1,R_2 = H	50.4%
**A:** Me_2NH	**S_2** : R_1,R_2 = Me	95.1%
**A:** $MeNH_2$	**S_3** : R_1 = Me, R_2 = H	59.7%
**A:** $PhNH_2$	**S_4** : R_1 = Ph, R_2 = H	52.5%
**A:** Bn_2NH	**S_5** : R_1,R_2 = Bn	58.5%
**A:** *p*-C_6H_{13}-NH_2	**S_6** : R_1 = *p*-C_6H_{13}-Ph, R_2 = H	95.0%

General procedure for amide synthesis (S1-6): To a solution of N-Boc-(3,3,3-trifluoro alanine) (1 equiv.), corresponding amine (1.2 molar equiv.), and N,N-diisopropyl ethylamine (2.5 molar equiv.) in methylene chloride was added HATU (1.2 molar equiv.). The reaction mixture was stirred at room temperature for 16 to 20 h, whereupon it was diluted with methylene chloride and washed with a saturated aqueous solution of sodium bicarbonate, water, and brine. The organic layer was dried ($MgSO_4$), filtered and concentrated under reduced pressure. Purification via silica gel flash column chromatography afforded amides **S_1-S_6**.

tert-butyl (3-amino-1,1,1-trifluoro-3-oxopropan-2-yl)carbamate, S1: White amorphous solid. ^{1}H NMR (400 MHz, DMSO-*d*$_6$) δ 7.87 (s, 1H, -NH_2), 7.66 (s, 1H, -NH_2), 7.58 (d, *J* = 9.1 Hz, 1H, -NHBoc), 4.87 (t, *J* = 9.1 Hz, 1H, -CH-), 1.41 (s, 9H, -$(CH_3)_3$). ^{13}C NMR (101 MHz, DMSO-*d*$_6$) δ 165.4, 155.3, 125.4, 80.0, 55.1, 28.3.

tert-butyl (3-(dimethylamino)-1,1,1-trifluoro-3-oxopropan-2-yl)carbamate, S2: White amorphous solid. ^{1}H NMR (400 MHz, Chloroform-*d*) δ 5.73 (d, *J* = 9.1 Hz, 1H,

-N$\underline{H}$Boc), 5.29 (p, *J* = 9.1, 7.0 Hz, 1H, -C$\underline{H}$-), 3.12 (s, 3H, -NHC$\underline{H}_3$), 3.01 (s, 3H, -NHC$\underline{H}_3$), 1.43 (s, 9H, -(C$\underline{H}_3$)$_3$). ^{13}C NMR (101 MHz, cdcl$_3$) δ 163.9, 154.7, 121.8, 81.0, 51.1, 36.2, 28.1.

tert-butyl (1,1,1-trifluoro-3-(methylamino)-3-oxopropan-2-yl)carbamate, S3: Sub-white amorphous solid. 1H NMR (400 MHz, Chloroform-d) δ 6.47 (s, 1H, -NHCH3), 5.70 (d, J = 8.5 Hz, 1H, -NHBoc), 4.86 (t, J = 8.5 Hz, 1H, -CH-), 2.87 (d, J = 4.8 Hz, 3H, -CH3), 1.44 (s, 9H, -(CH3)3). 13C NMR (101 MHz, Chloroform-d) δ 163.7, 155.0, 124.4, 81.3, 55.4, 28.1, 26.7.

tert-butyl (1,1,1-trifluoro-3-oxo-3-(phenylamino)propan-2-yl)carbamate, S4: Sub-yellow amorphous solid. ^{1}H NMR (400 MHz, Chloroform-*d*) δ 8.42 – 8.33 (m, 1H, -N$\underline{H}$Ph), 7.47 (dd, *J* = 8.6, 1.2 Hz, 2H, *o*-C_6H_5), 7.37 – 7.27 (m, 2H, *m*-C_6H_5), 7.19 – 7.11 (m, 1H, *p*-C_6H_5), 5.89 (d, *J* = 9.2 Hz, 1H, -N$\underline{H}$Boc), 5.16 (t, *J* = 8.1 Hz, 1H, -C$\underline{H}$-), 1.45 (s, 9H, -(C$\underline{H}_3$)$_3$). ^{13}C NMR (101 MHz, Chloroform-*d*) δ 161.7, 155.3, 136.5, 129.0, 125.4, 121.6, 120.6, 81.7, 56.1, 28.1.

tert-butyl (3-(dibenzylamino)-1,1,1-trifluoro-3-oxopropan-2-yl)carbamate, S5: Yellow amorphous solid. ^{1}H NMR (400 MHz, Chloroform-*d*) δ 7.42 – 7.27 (m, 6H, -C_6H_5), 7.20 – 7.07 (m, 4H, -C_6H_5), 5.76 (d, *J* = 9.2 Hz, 1H, -C$\underline{H}_2$Ph), 5.45 (t, *J* = 9.2, 6.5 Hz, 1H, -C$\underline{H}_2$Ph), 5.09 (d, *J* = 14.9 Hz, 1H, -N$\underline{H}$Boc), 4.71 (d, *J* = 16.7 Hz, 1H, -C$\underline{H}_2$Ph), 4.34 (d, *J* = 16.7 Hz, 1H, -C$\underline{H}_2$Ph), 4.09 (d, *J* = 14.9 Hz, 1H, -C$\underline{H}$-), 1.43 (s, 9H, -(C$\underline{H}_3$)$_3$). ^{13}C NMR (101 MHz, Chloroform-*d*) δ 154.4, 135.8, 129.0, 128.7, 128.3, 128.1, 127.9, 127.7, 127.0, 81.1, 50.2, 48.2, 28.1.

***tert*-butyl (1,1,1-trifluoro-3-((4-hexylphenyl)amino)-3-oxopropan-2-yl)carbamate S6:** Yellow amorphous solid. ^{1}H NMR (400 MHz, Chloroform-*d*) δ 7.99 (s, 1H, -N$\underline{H}$Ph), 7.37 (d, *J* = 8.5 Hz, 2H, -*o*-$C_6\underline{H}_4$NH-), 7.12 (d, *J* = 8.4 Hz, 2H, -*m*-$C_6\underline{H}_4$NH-), 5.78 (d, *J* = 9.0 Hz, 1H, -N$\underline{H}$Boc), 5.05 (s, 1H, -C$\underline{H}$-), 2.64 – 2.44 (m, 2H, -$C\underline{H}_2(C_5H_{11})$), 1.73 – 1.51 (m, 2H, -$CH_2C\underline{H}_2(C_4H_9)$), 1.45 (s, 9H, -$(C\underline{H}_3)_3$), 1.29 – 1.24 (m, 6H, -$(CH_2)_2(C\underline{H}_2)_3CH_3$), 0.89 – 0.82 (m, 3H, -$C_5H_{10}C\underline{H}_3$). ^{13}C NMR (101 MHz, Chloroform-*d*) δ 161.3, 155.1, 140.4, 134.0, 128.9, 120.5, 118.6, 81.6, 56.0, 35.3, 31.6, 31.3, 28.8, 28.1, 22.5, 14.0.

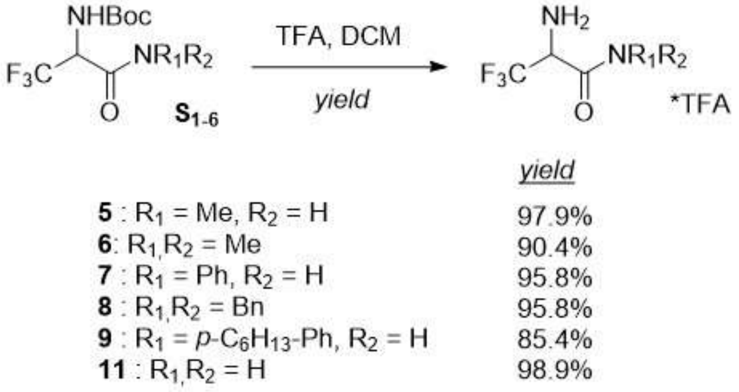

General procedure for Boc deprotection: To a solution of the corresponding protected amide in dry DCM, excess TFA (>100 equiv.) was added and stirred for 1 h at r.t.. Then excess reagent and solvent were removed under vacuum. The residue was dissolved in DCM and evaporated (x3) to obtain the product as a TFA salt.

2-amino-3,3,3-trifluoro-N-methylpropanamide, (5): White amorphous solid. ^{1}H NMR (400 MHz, DMSO-d_6) δ 8.68 (t, *J* = 8.8 Hz, 1H, -CON$\underline{H}$Me), 4.60 (q, *J* = 7.8 Hz, 1H,

-C$\underline{H}$-), 2.68 (d, *J* = 4.7 Hz, 3H, -NHC$\underline{H}_3$), 1.36 (s, 2H, -N$\underline{H}_2$). ^{13}C NMR (101 MHz, DMSO-d_6) δ 162.3, 124.7, 53.6, 26.3.

HR-MS (ESI-TOF) m/z: Calcd for $C_4H_8N_2OF_3$ 157.0589; Found $[M+H]^+$ 157.0590

2-amino-3,3,3-trifluoro-N,N-dimethylpropanamide, (6): White amorphous solid. ^{1}H NMR (400 MHz, DMSO-d_6) δ 5.40 (s, 1H, -C$\underline{H}$-), 3.07 (s, 3H, -N(C$\underline{H}_3$)$_2$), 2.91 (s, 3H, -N(C$\underline{H}_3$)$_2$) (-N$\underline{H}_2$ not visible due to deuterium exchange, or overlap with solvent peak). ^{13}C NMR (101 MHz, DMSO-d_6) δ 162.3, 121.5, 56.8, 36.4.

HR-MS (ESI-TOF) m/z: Calcd for $C_5H_{10}N_2OF_3$ 171.0745; Found $[M+H]^+$ 171.0749

2-amino-3,3,3-trifluoro-N-phenylpropanamide, (7): Yellowish amorphous solid. ^{1}H NMR (400 MHz, DMSO-d_6) δ 10.79 (s, 1H, -CON$\underline{H}$Ph), 7.56 (ddd, *J* = 8.7, 5.6, 1.1 Hz, 2H, *o*-C_6H_5), 7.36 (ddd, *J* = 8.7, 5.6, 1.9 Hz, 2H, *m*-C_6H_5), 7.14 (dt, *J* = 12.6, 3.7 Hz, 1H, *p*-C_6H_5), 4.72 (q, *J* = 7.7 Hz, 1H, -C$\underline{H}$-), 1.38 (s, 2H, -N$\underline{H}_2$). ^{13}C NMR (101 MHz, DMSO-d_6) δ 166.2, 137.9, 129.5, 129.3, 125.1, 120.0, 55.1.

HR-MS (ESI-TOF) m/z: Calcd for $C_3H_{10}N_2OF_3$ 219.0745; Found $[M+H]^+$ 219.0751

2-amino-N,N-dibenzyl-3,3,3-trifluoropropanamide, (8): Sub-yellow amorphous solid. ^{1}H NMR (400 MHz, DMSO-d_6) δ 7.46 – 6.99 (m, 10H, (-C_6H_5)$_2$), 5.72 (d, *J* = 1.1 Hz, 1H, -C$\underline{H}$-), 4.88 (d, *J* = 16.2 Hz, 1H, -C$\underline{H}_2$Ph), 4.76 (d, *J* = 15.1 Hz, 1H, -C$\underline{H}_2$Ph), 4.41 (d, *J* = 16.2 Hz, 1H, -C$\underline{H}_2$Ph), 4.10 (d, *J* = 15.1 Hz, 1H, -C$\underline{H}_2$Ph) (-N$\underline{H}_2$ not visible due to deuterium exchange, , or overlap with solvent peak). ^{13}C NMR (101 MHz, DMSO-d_6) δ 163.0, 136.5, 136.1, 129.1, 128.9, 128.2, 127.9, 55.3, 48.3.

HR-MS (ESI-TOF) m/z: Calcd for $C_{17}H_{18}N_2OF_3$ 323.1371; Found $[M+H]^+$ 323.1377

2-amino-3,3,3-trifluoro-N-(4-hexylphenyl)propanamide, (9): Sub-yellow amorphous solid. ^{1}H NMR (400 MHz, DMSO-*d*$_6$) δ 10.73 (s, 1H, -CON<u>H</u>Ph), 7.46 (d, *J* = 8.5 Hz, 1H, -*o*-$C_6\underline{H}_4$NH-), 7.16 (d, *J* = 8.5 Hz, 1H, -*m*-$C_6\underline{H}_4$NH-), 4.71 (dd, *J* = 15.3, 7.6 Hz, 1H, -C<u>H</u>-), 2.50 (dd, *J* = 12.9, 5.1 Hz, 2H, -$C\underline{H}_2(C_5H_{11})$), 1.61 – 1.40 (m, 2H, -$CH_2C\underline{H}_2(C_4H_9)$), 1.32 – 1.16 (m, 6H, -$(CH_2)_2(C\underline{H}_2)_3CH_3$), 0.81 (t, *J* = 6.8 Hz, 3H, -$C_5H_{10}C\underline{H}_3$). ^{13}C NMR (101 MHz, DMSO-*d*$_6$) δ 160.8, 139.3, 135.6, 129.2, 122.0, 120.0, 54.9, 34.9, 31.5, 28.6, 22.4, 14.3.

HR-MS (ESI-TOF) m/z: Calcd for $C_{15}H_{22}N_2OF_3$ 303.1684; Found $[M+H]^+$ 303.1693

2-amino-3,3,3-trifluoropropanamide, (11): White amorphous solid. ^{1}H NMR (400 MHz, DMSO-*d*$_6$) δ 8.16 (s, 1H, -$CON\underline{H}_2$), 8.02 (s, 1H, -$CON\underline{H}_2$), 4.66 (q, *J* = 7.9 Hz, 1H, -C<u>H</u>-) (-$N\underline{H}_2$ not visible due to deuterium exchange, or overlap with solvent peak). ^{13}C NMR (101 MHz, DMSO-*d*$_6$) δ 163.1, 124.4, 54.0.

HR-MS (ESI-TOF) m/z: Calcd for $C_3H_4N_2O_2F_3$ 157.0225; Found $[M-H+OH]^-$ 157.0227

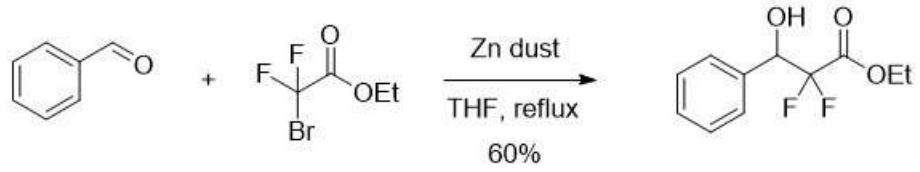

ethyl 2,2- difluoro- 3- hydroxy- 3- phenylpropanoate, (S7): Zinc dust was suspended to THF and the suspension was heated to reflux. Ethyl bromodifluoroacetate was then added to the suspension and within 1 min benzaldehyde was added and refluxing continued for 15 min. the reaction mixture was cooled to r.t. and then poured into a mixture of AcOEt, 1M aq. $KHSO_4$ and sat. $NaHCO_3$. the whole was stirred for 15 min and the layers were separated and the aqueous layer was extracted with AcOEt. the combined organic layers were dried and the solvent evaporated. Purification via silica gel flash column chromatography afforded bio-052 as colorless oil (60.3%).

Spectroscopic characterization matched with literature data, *Chem. Pharm. Bull.* ***45****(11), 1793-1804 (1997)*

^{1}H NMR (400 MHz, $CDCl_3$) δ 7.45 – 7.34 (m, 5H, -C_6H_5), 5.15 (dd, J = 15.1, 7.9 Hz, 1H, -C<u>H</u>(OH)-), 4.29 (q, J = 7.1 Hz, 2H, -C<u>H2</u>CH_3), 2.69 (s, 1H, -OH), 1.27 (t, J = 7.1 Hz, 3H, -CH_2C<u>H3</u>).

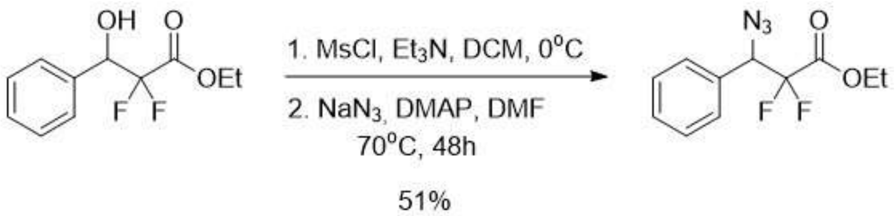

ethyl 3-azido-2,2-difluoro-3-phenylpropanoate, (S8): Mesyl chloride was added dropwise to a stirred solution of bio-052 and trimethylamine in DCM at 0°C. The reaction mixture was stirred overnight, then washed with 10% HCl, water, sat. aq. $NaHCO_3$ and brine. The organic layer was dried over anhydrous $MgSO_4$ and the solvent was removed under reduced pressure. The residue was dissolved in DMF and DMAP

and NaN_3 were added at r.t. The mixture was heated to 70°C for 48 hours. It was then poured into ice-water. The aqueous phase was extracted with AcOEt and the organic layer was washed with water, dried over $MgSO_4$ and the solvent was removed under reduced pressure. Purification via silica gel flash column chromatography afforded bio-053 as colorless oil (51%). Spectroscopic characterization matched with literature data, *Chem. Pharm. Bull.* ***45****(11), 1793-1804 (1997)*

^1H NMR (400 MHz, $CDCl_3$) δ 7.47 – 7.36 (m, 5H, $-C_6H_5$), 5.08 (dd, J = 14.8, 10.3 Hz, 1H, $-C\underline{H}(N_3)-$), 4.30 (q, J = 7.1 Hz, 2H, $-C\underline{H_2}CH_3$), 1.28 (t, J = 7.1 Hz, 3H, $-CH_2C\underline{H_3}$).

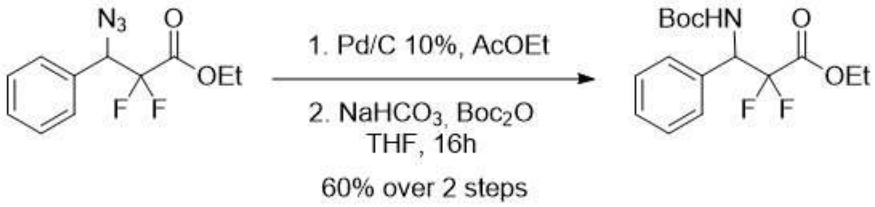

ethyl 3-{[(tert-butoxy)carbonyl] amino}-2,2-difluoro-3-phenylpropanoate (S9): A mixture of bio-053 and 10% pd/c in AcOEt was shaken in hydrogen atmosphere at r.t. until full consumption of the s.m. The catalyst was removed by filtration and the filtrate was concentrated under reduced pressure. The residue was taken up in dry THF, then $NaHCO_3$ and Boc_2O were added at r.t. The reaction mixture was stirred at 60°C for 17h, diluted with AcOEt and washed with water, 10% HCl, sat. $NaHCO_3$ and brine. The organic layer was dried over anhydrous $MgSO_4$ and the solvent removed under reduced pressure. The residue was purified by silica gel column chromatography to obtain bio-054 as colorless solid (60% over 2 steps).

Spectroscopic characterization matched with literature data, *Chem. Pharm. Bull.* ***45****(11), 1793-1804 (1997)*

^1H NMR (400 MHz, $CDCl_3$) δ 7.40 – 7.28 (m, 5H, $-C_6H_5$), 5.49 – 5.23 (m, 2H, -

CH(NHBoc)-), 4.31 – 4.16 (m, 2H, -CH_2CH_3), 1.40 (s, 9H), 1.24 (t, J = 7.1 Hz, 3H, -CH_2CH_3).

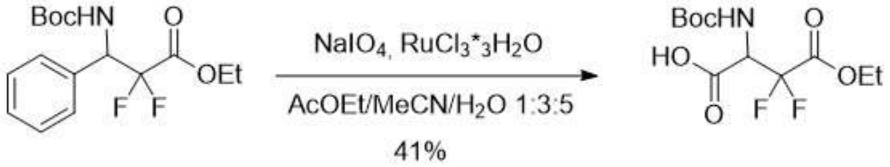

2-{[(tert-butoxy) carbonyl] amino}-4-ethoxy-3,3-difluoro-4-oxobutanoic acid, (S10): To a solution of bio-054 (1 equiv.) in AcOEt/CH_3CN/H_2O (1:3:5) was added $NaIO_4$ (18.0 equiv.) at room temperature. $RuCl_3$*$3H_2O$ (2.0 mol%) was introduced (in 2 parts) to this mixture which was stirred for 1 day at room temperature. After filtration, saturated aq. $NaHCO_3$ was added in order to adjust the pH of the solution to 8–9, and the aqueous layer was washed with CH_2Cl_2. Then, 1 M HCl was added to this aqueous layer to adjust the pH at 2–3, which was extracted with EtOAc twice, and the organic layer was washed with brine, and dried over anhydrous Na_2SO_4. Filtration and concentration followed by silica gel column chromatography furnished bio-055 as subwhite solid (41%).

^{1}H NMR (300 MHz, MeOD) δ 5.08 (dd, J = 20.3, 8.5 Hz, 1H, -CH(NHBoc)-), 4.37 (q, J = 7.1 Hz, 2H, -CH_2CH_3), 3.37 – 3.32 (m, 1H, -CH(NHBoc)-), 1.49 (s, 9H, -$(CH_3)_3$), 1.36 (t, J = 7.1 Hz, 3H, -CH_2CH_3). ^{13}C NMR (101 MHz, MeOD) δ 167.67, 162.45, 156.32, 112.92, 79.90, 62.79, 56.00, 27.13, 12.67.

In HRMS conditions no signal observed

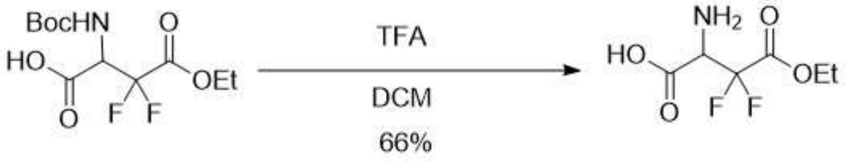

3-{[(tert-butoxy) carbonyl] amino}-2,2-difluorobutanedioic acid, (13): To a solution of bio-055 in dry DCM, excess TFA (>100 equiv.) was added and stirred for 1 h at r.t. Then excess reagent and solvent were removed under vacuum. The residue was dissolved in DCM and evaporated (x3) to obtain bio-092 as TFA salt (66%).

^{1}H NMR (300 MHz, MeOD) δ 4.44 (dd, *J* = 25.5, 3.5 Hz, 1H, -C$\underline{H}$(NH_2)-), 4.28 (q, *J* = 7.1 Hz, 2H, -C$\underline{H_2}$$CH_3$), 1.26 (t, *J* = 7.1 Hz, 3H, -CH_2C$\underline{H_3}$). ^{13}C NMR (101 MHz, MeOD) δ 164.55, 161.26, 112.93, 63.01, 57.06, 12.57. HR-MS (ESI-TOF) m/z: Calcd for $C_6H_{10}NO_4F_2$ 198.0578; Found $[M+H]^+$ 198.0583

NMR Spectra of final products

^{1}H NMR of **2-amino-3,3,3-trifluoro-N-hydroxypropanamide (5)** in DMSO-d_6:

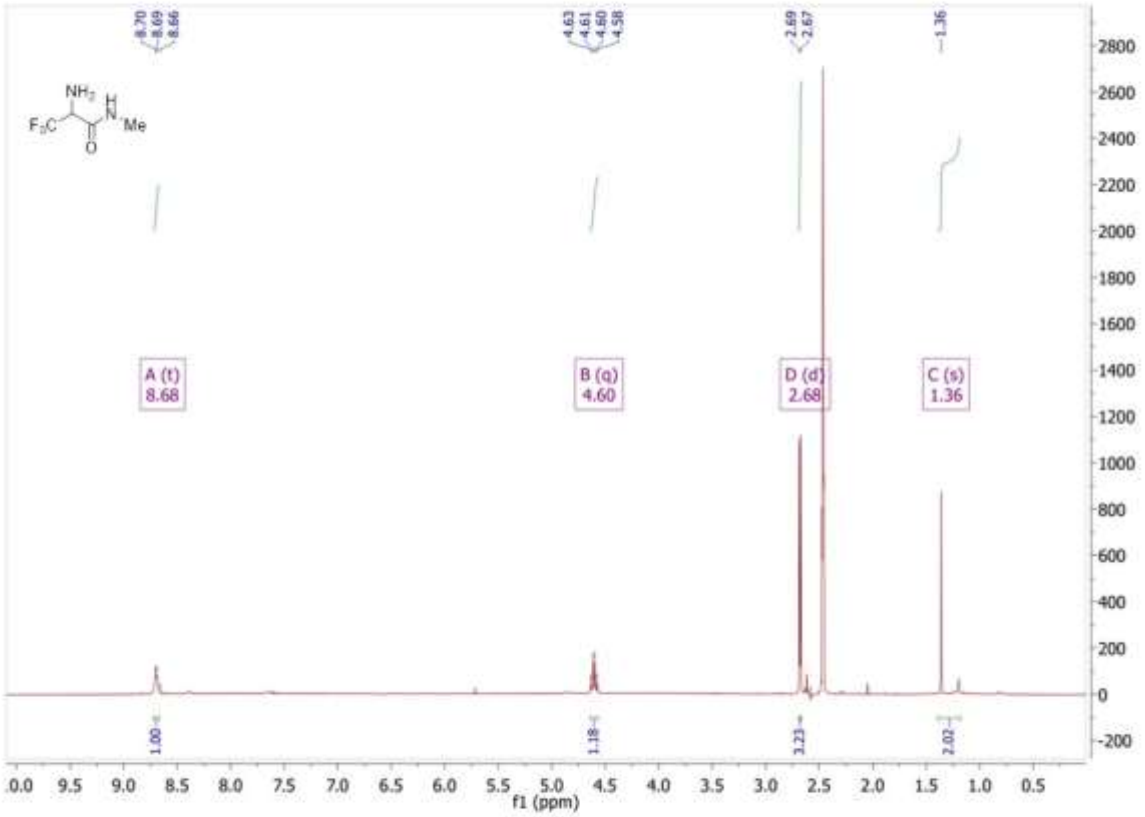

^{13}C NMR of **2-amino-3,3,3-trifluoro-N-hydroxypropanamide (5)** in DMSO-d_6:

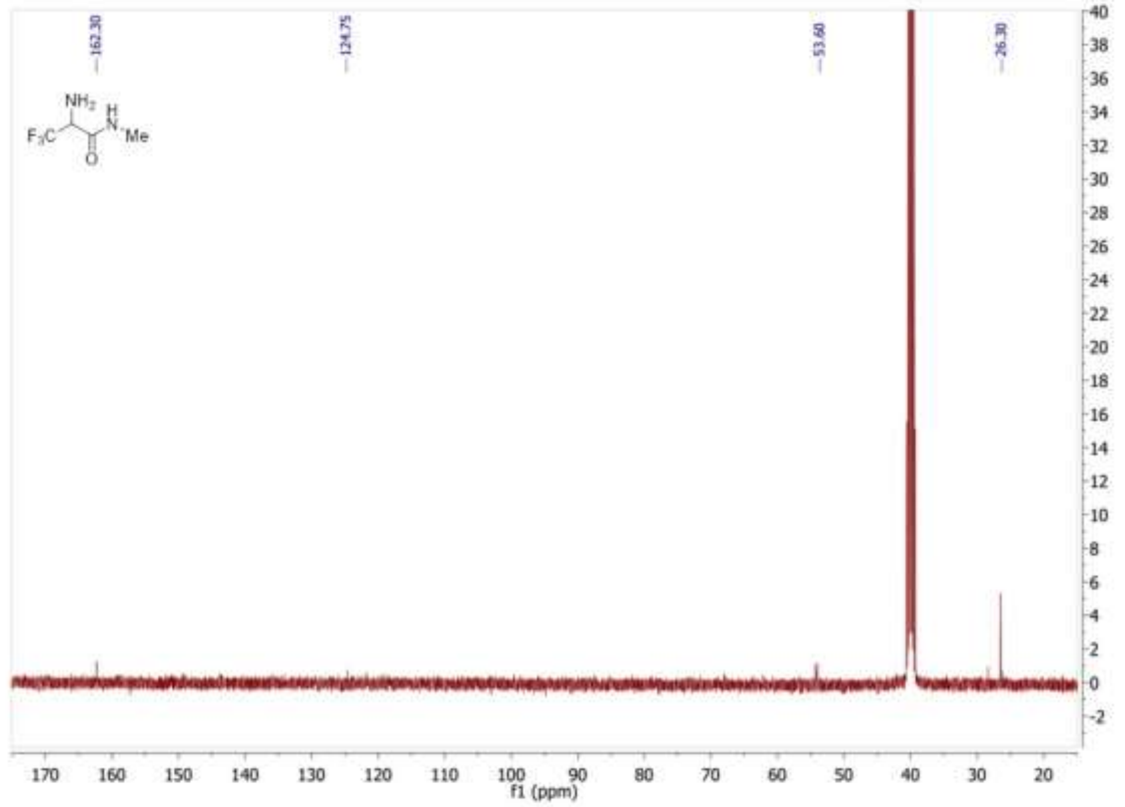

^{1}H NMR of **2-amino-3,3,3-trifluoropropanamide (6)** in DMSO-d_6:

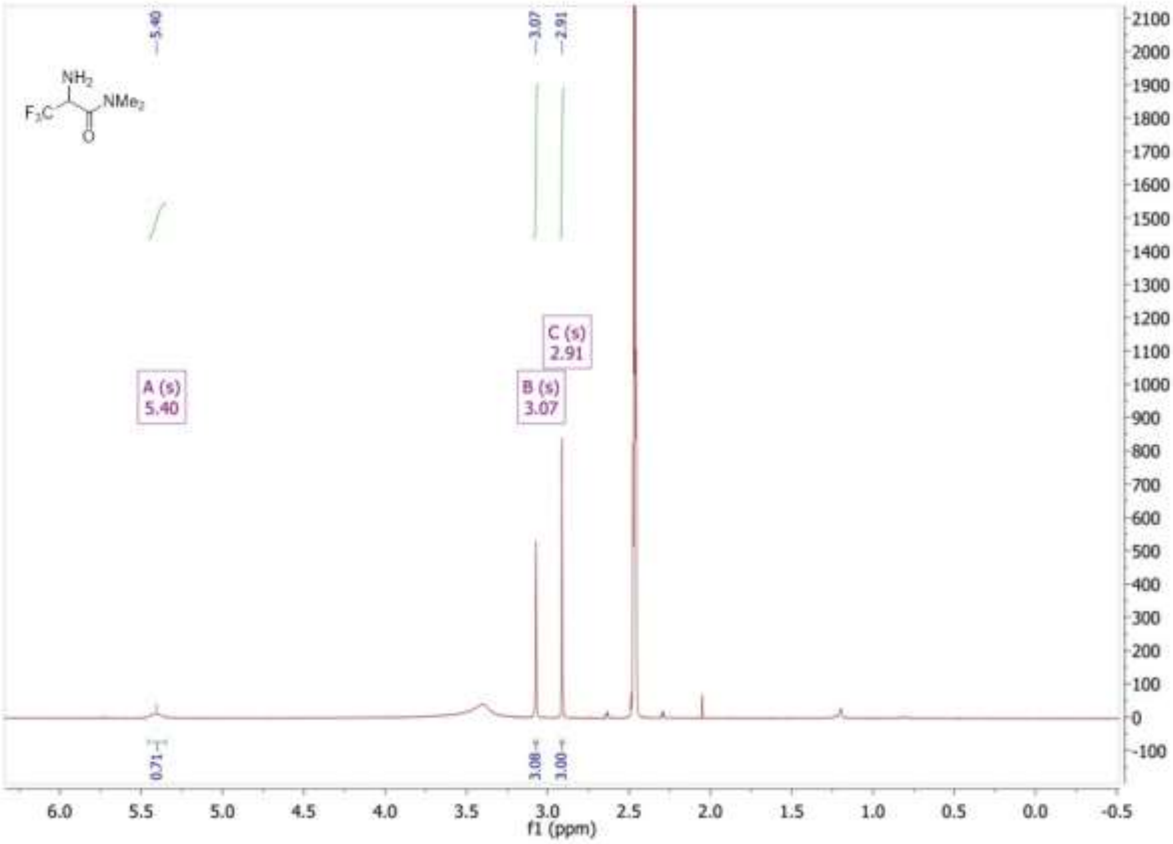

^{13}C NMR of **2-amino-3,3,3-trifluoropropanamide (6)** in DMSO-d_6:

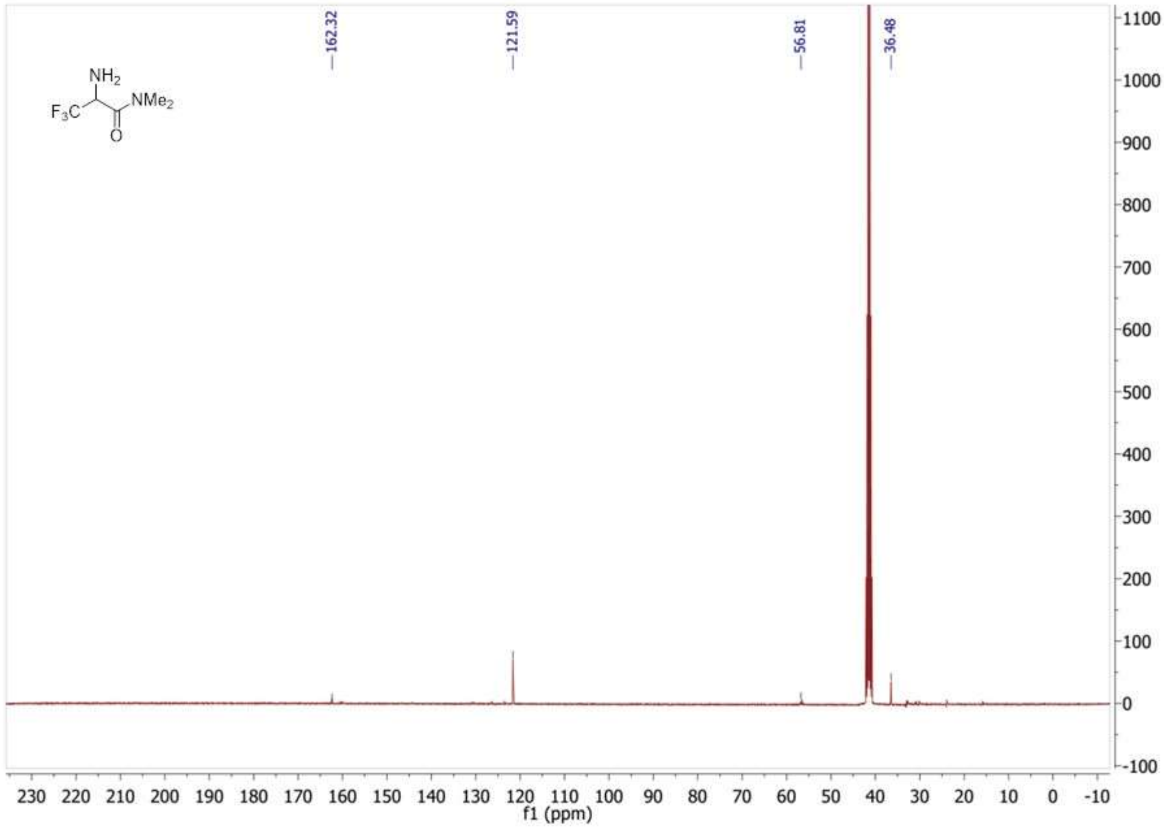

^{1}H NMR of **2-amino-3,3,3-trifluoro-N,N-dimethylpropanamide (7)** in DMSO-d_6:

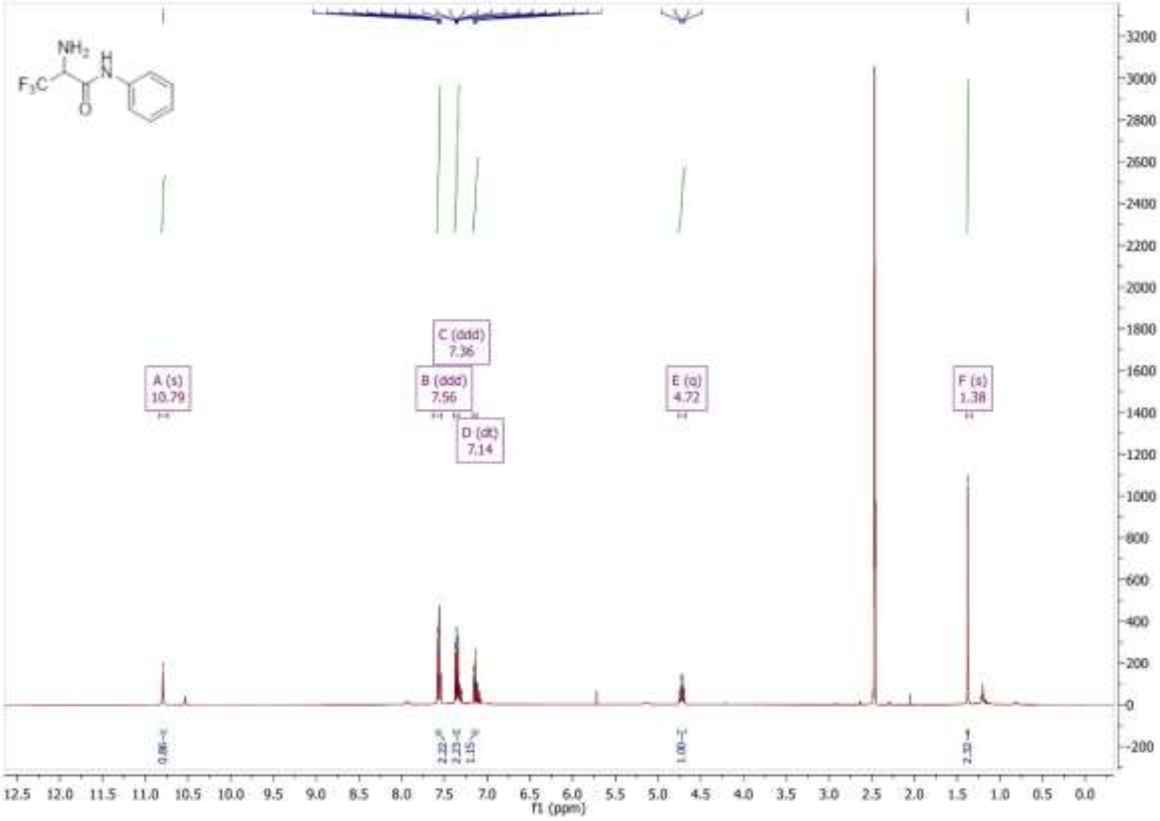

^{13}C NMR of **2-amino-3,3,3-trifluoro-N,N-dimethylpropanamide (7)** in DMSO-d_6:

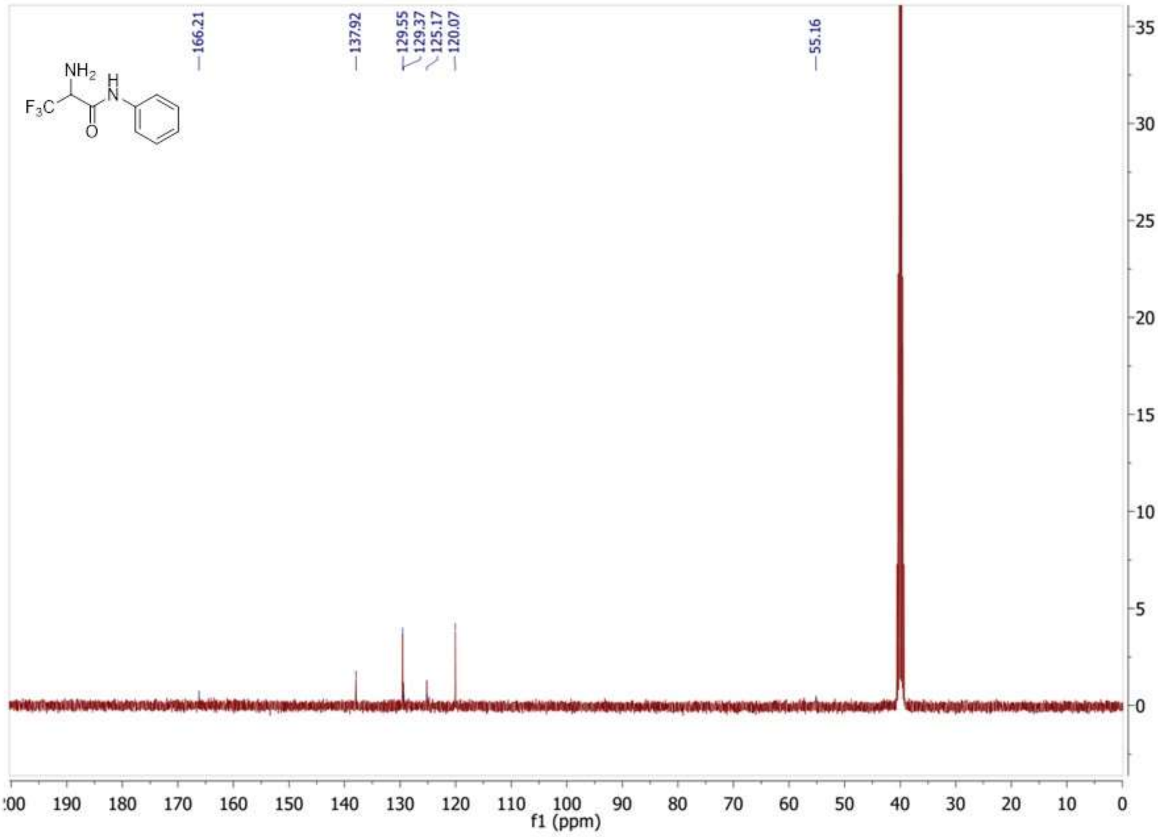

^{1}H NMR of **2-amino-3,3,3-trifluoro-N-methylpropanamide (8)** in DMSO-d_6:

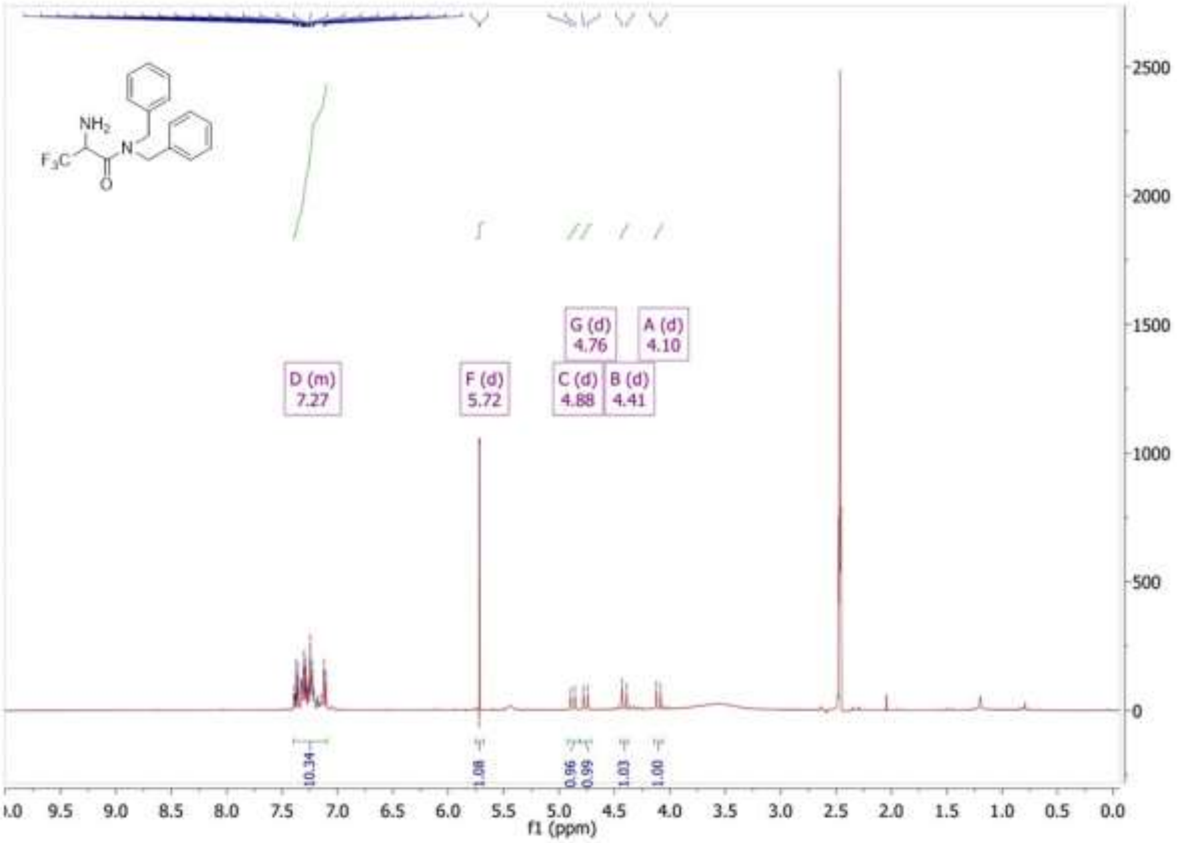

^{13}C NMR of **2-amino-3,3,3-trifluoro-N-methylpropanamide (8)** in DMSO-d_6:

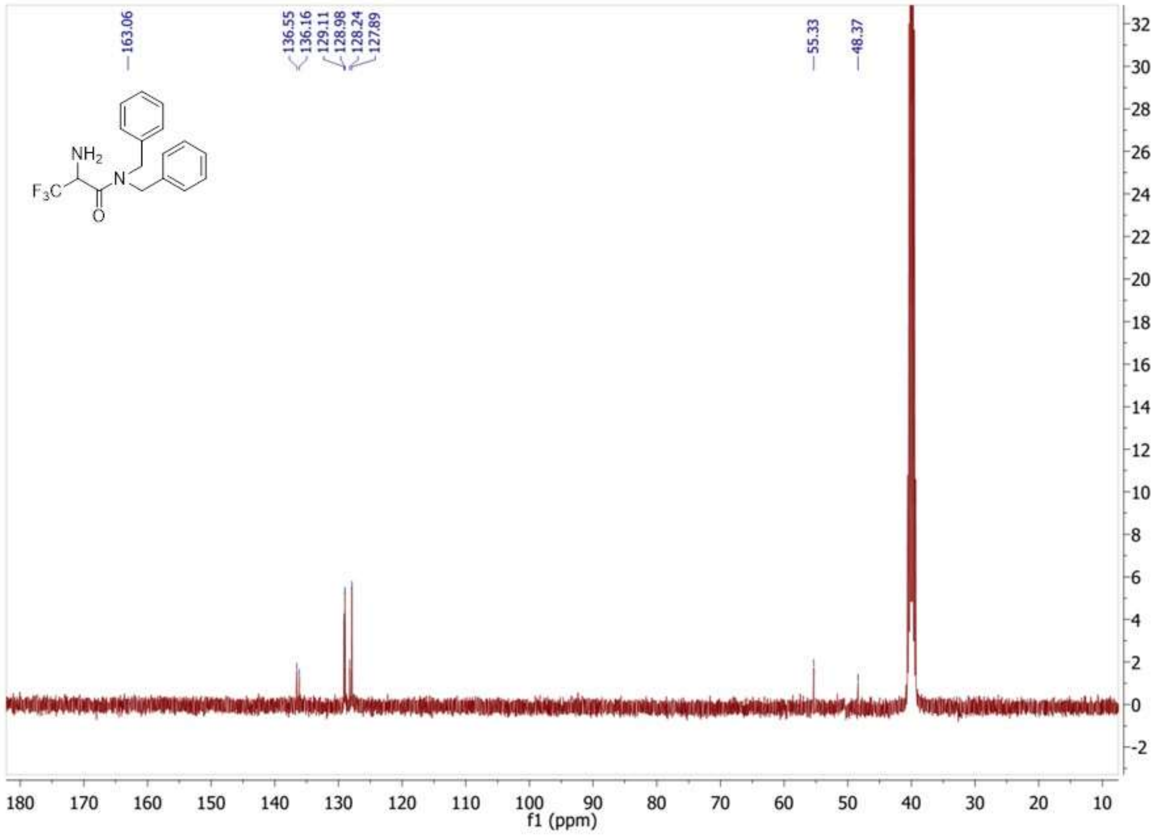

^{1}**H NMR** of **2-amino-3,3,3-trifluoro-N-phenylpropanamide (9)** in DMSO-d_6:

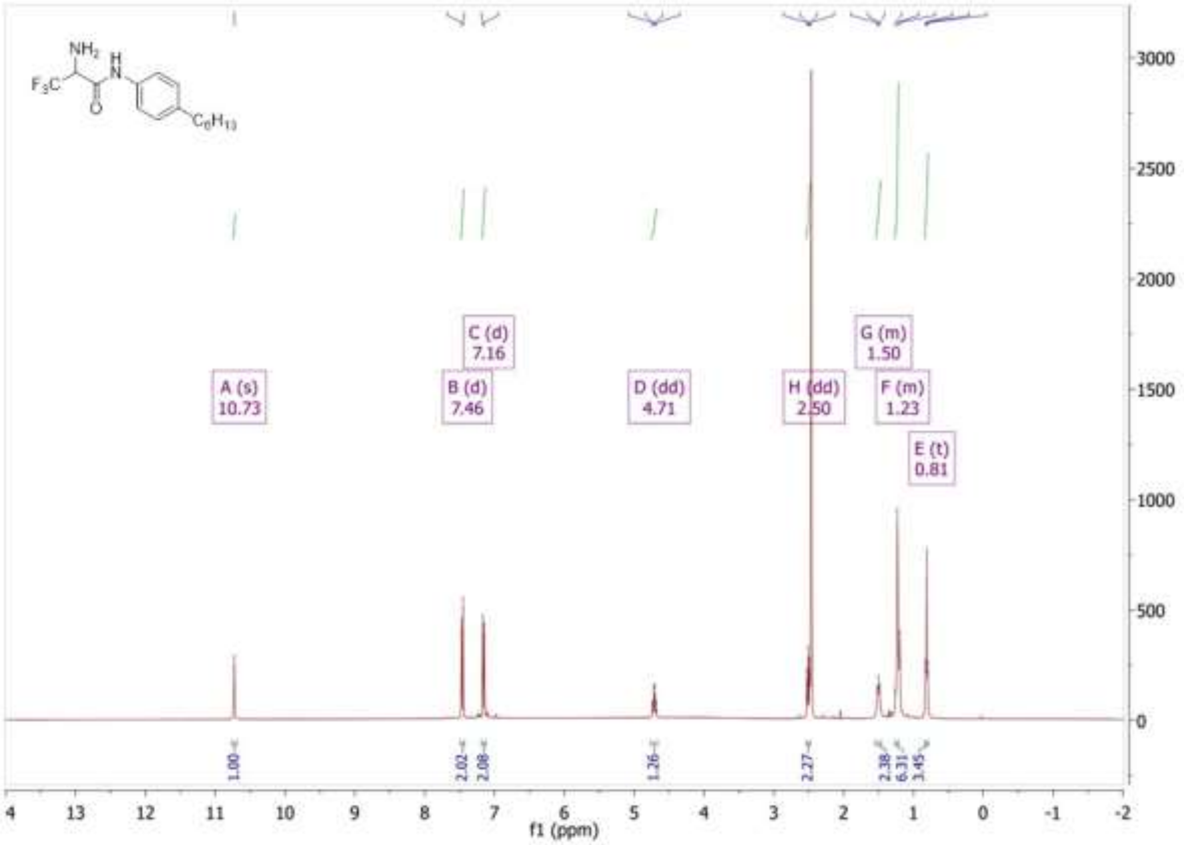

^{13}**C NMR** of **2-amino-3,3,3-trifluoro-N-phenylpropanamide (9)** in DMSO-d_6:

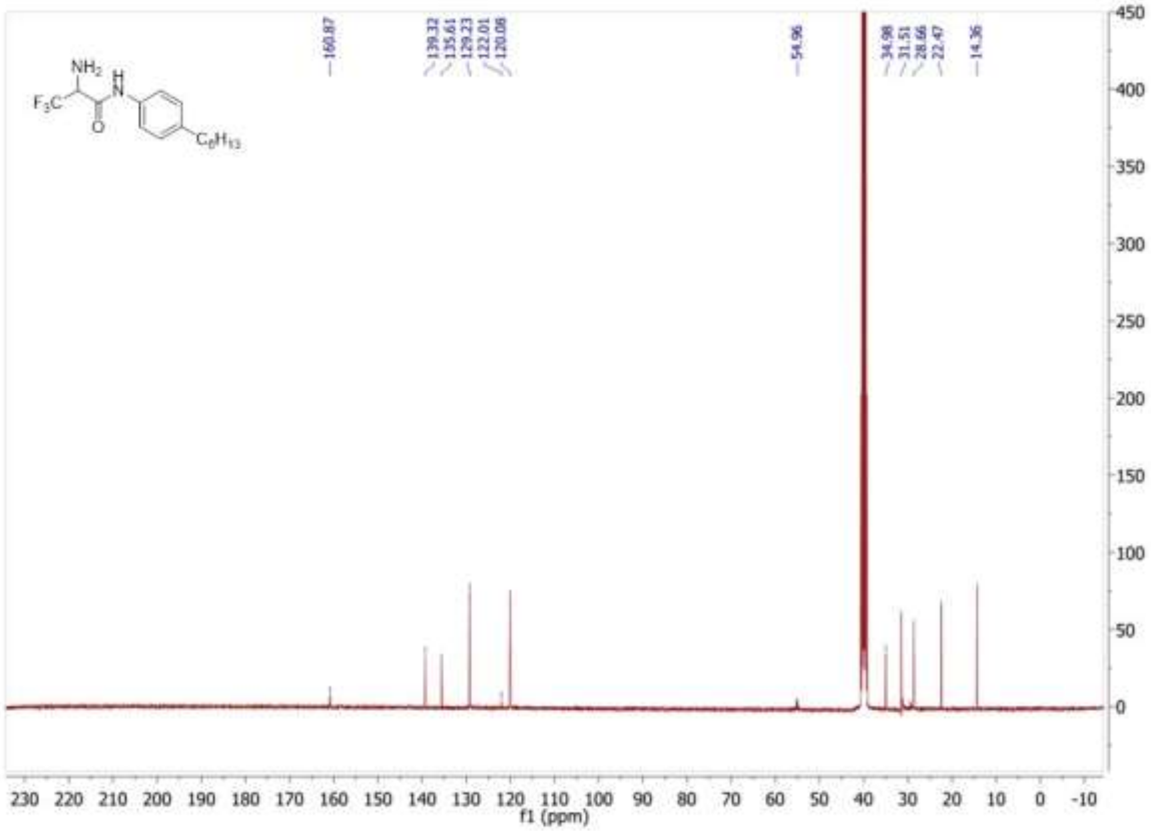

^{1}H NMR of **2-amino-N,N-dibenzyl-3,3,3-trifluoropropanamide (10)** in DMSO-d_6:

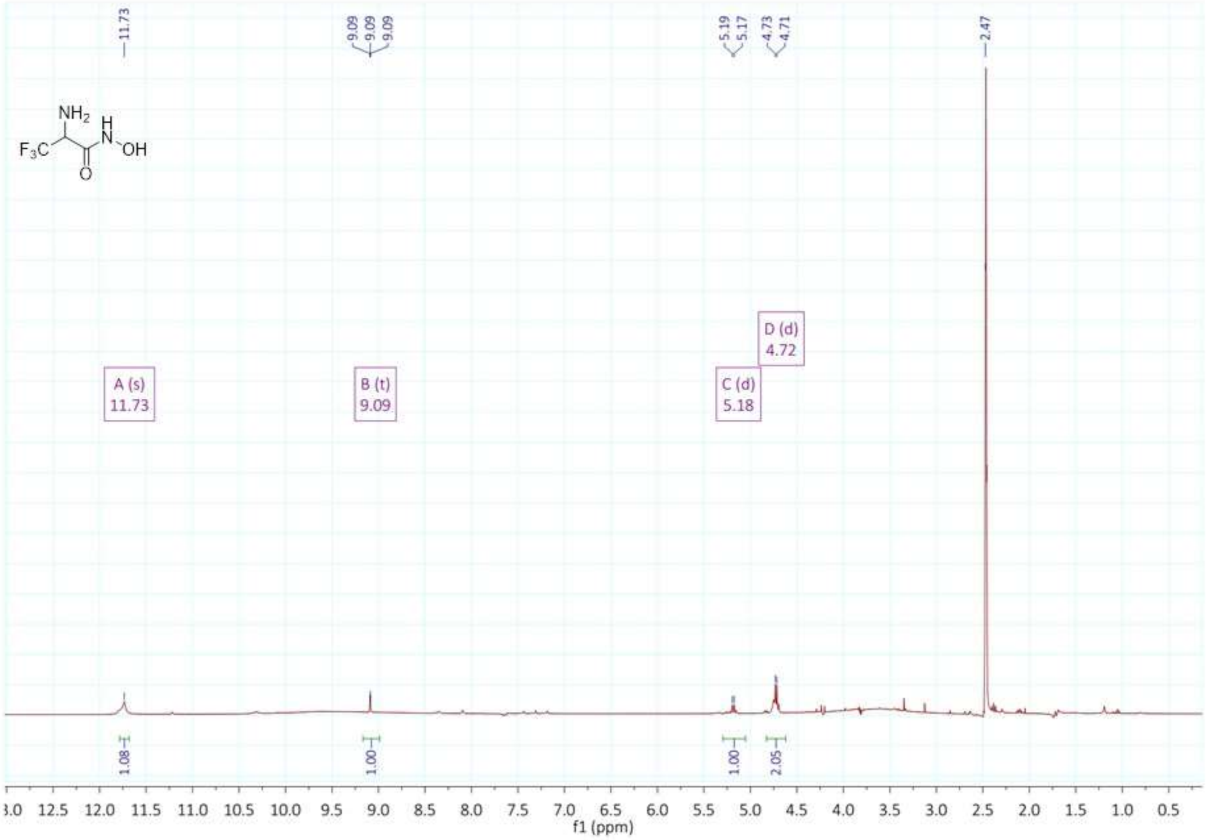

^{13}C NMR of **2-amino-N,N-dibenzyl-3,3,3-trifluoropropanamide (10)** in DMSO-d_6:

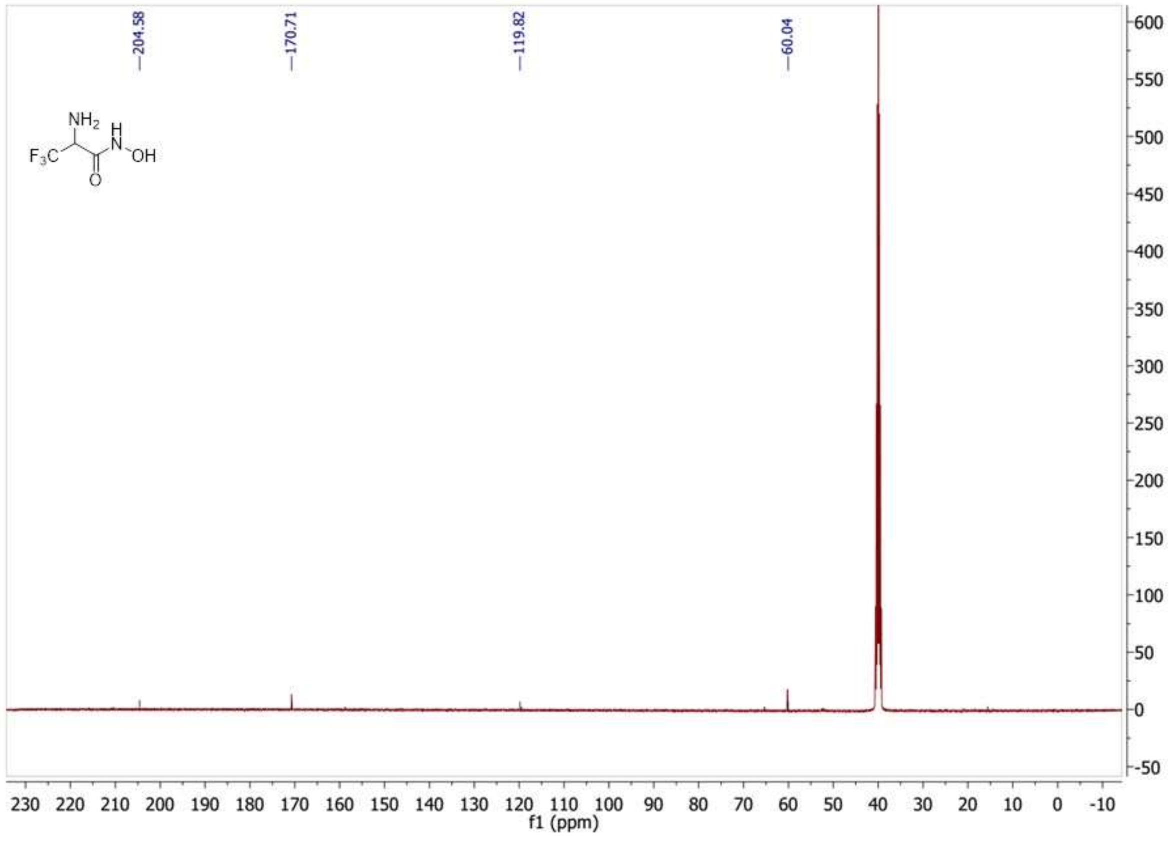

^{1}**H NMR** of **2-amino-3,3,3-trifluoro-N-(4-hexylphenyl)propanamide (11)** in DMSO-d_6:

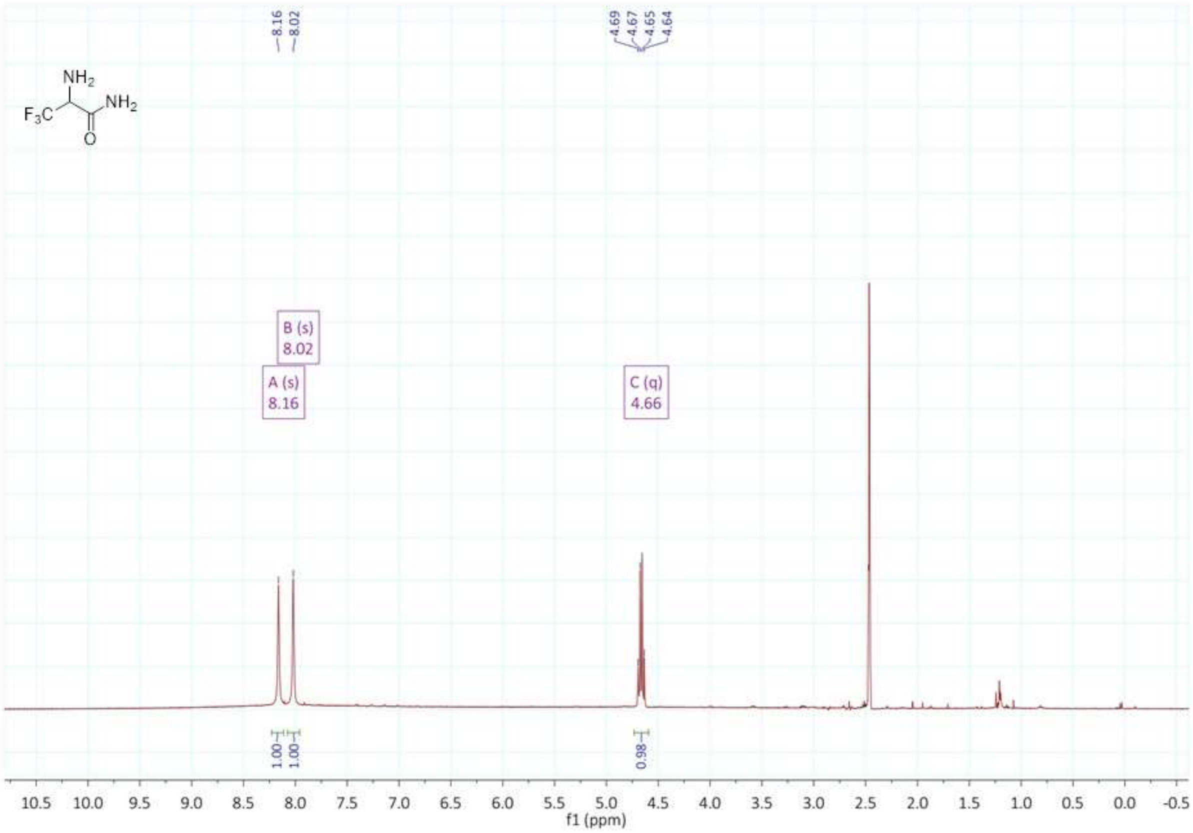

^{13}**C NMR** of **2-amino-3,3,3-trifluoro-N-(4-hexylphenyl)propanamide (11)** in DMSO-d_6:

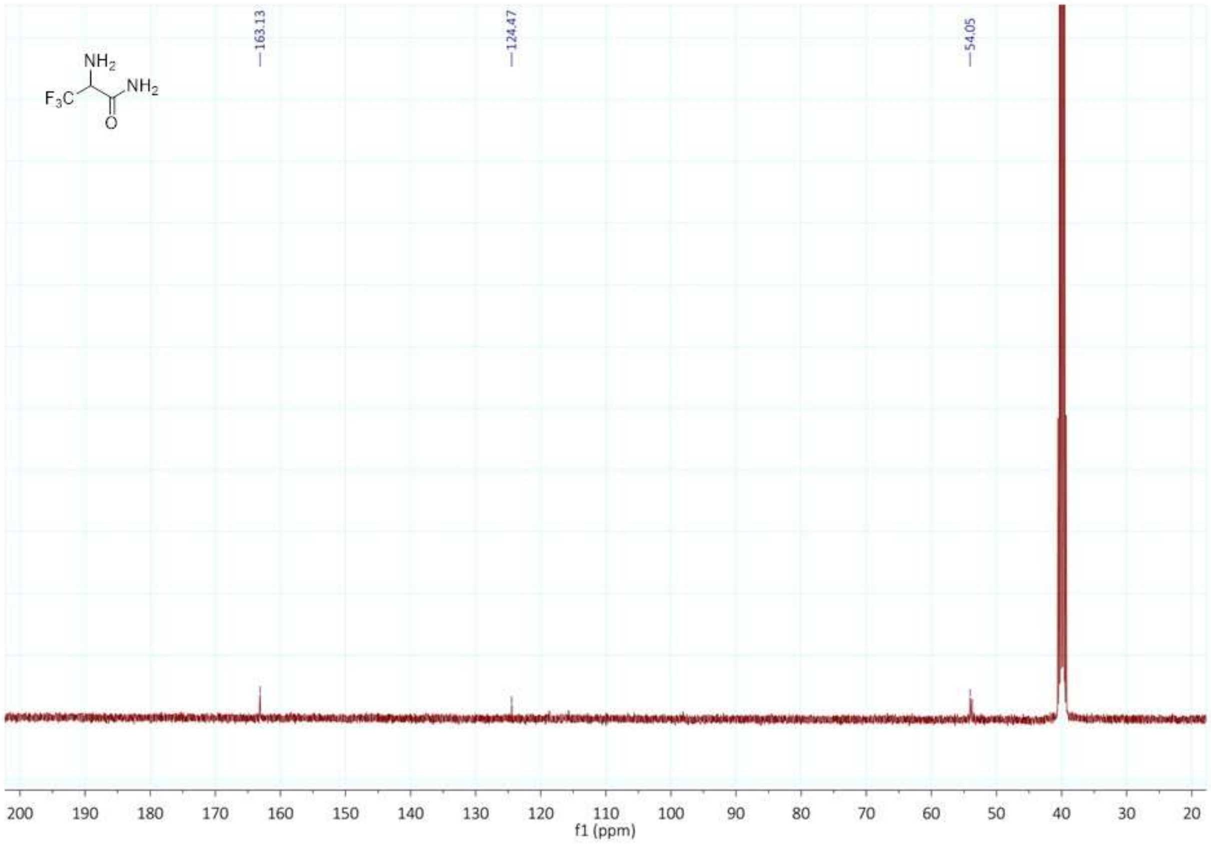

^{1}H NMR of **2-amino-3,3,3-trifluoro-N-(4-hexylphenyl)propanamide (13)** in DMSO-d_6:

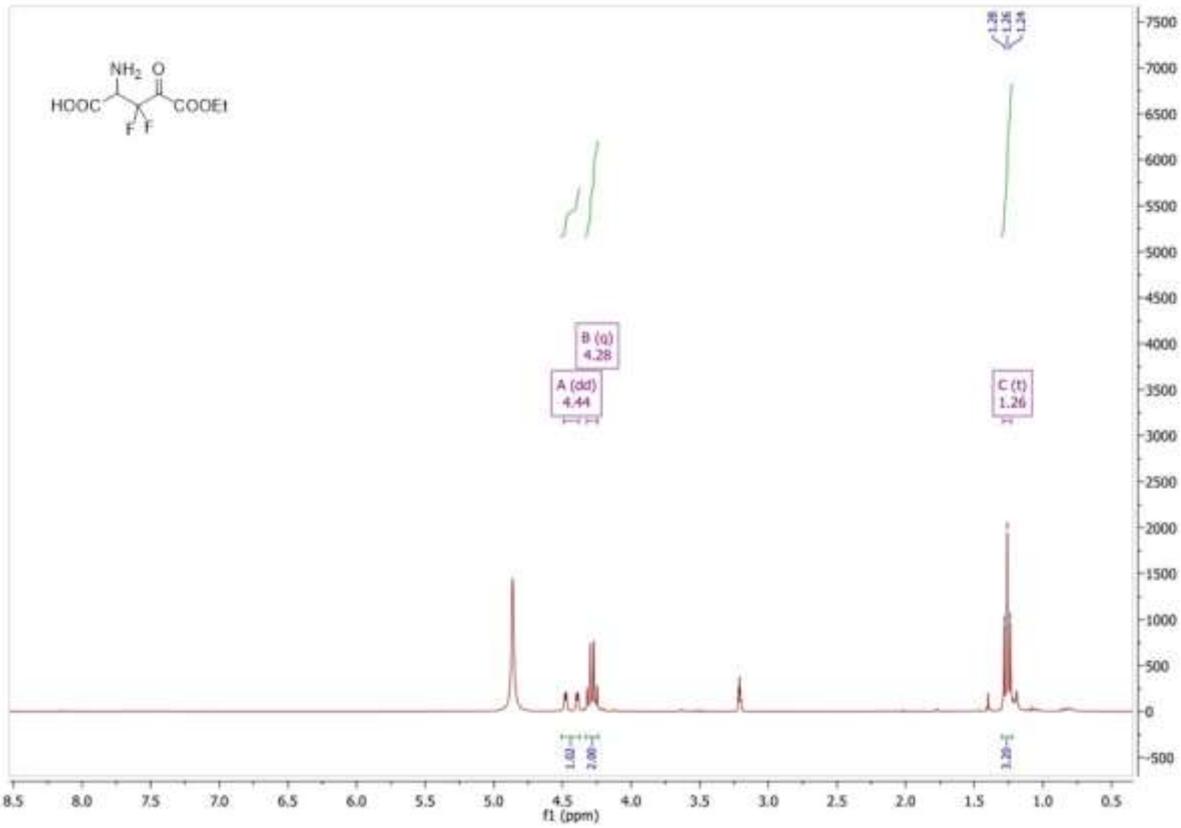

^{13}C NMR of **2-amino-3,3,3-trifluoro-N-(4-hexylphenyl)propanamide (13)** in DMSO-d_6:

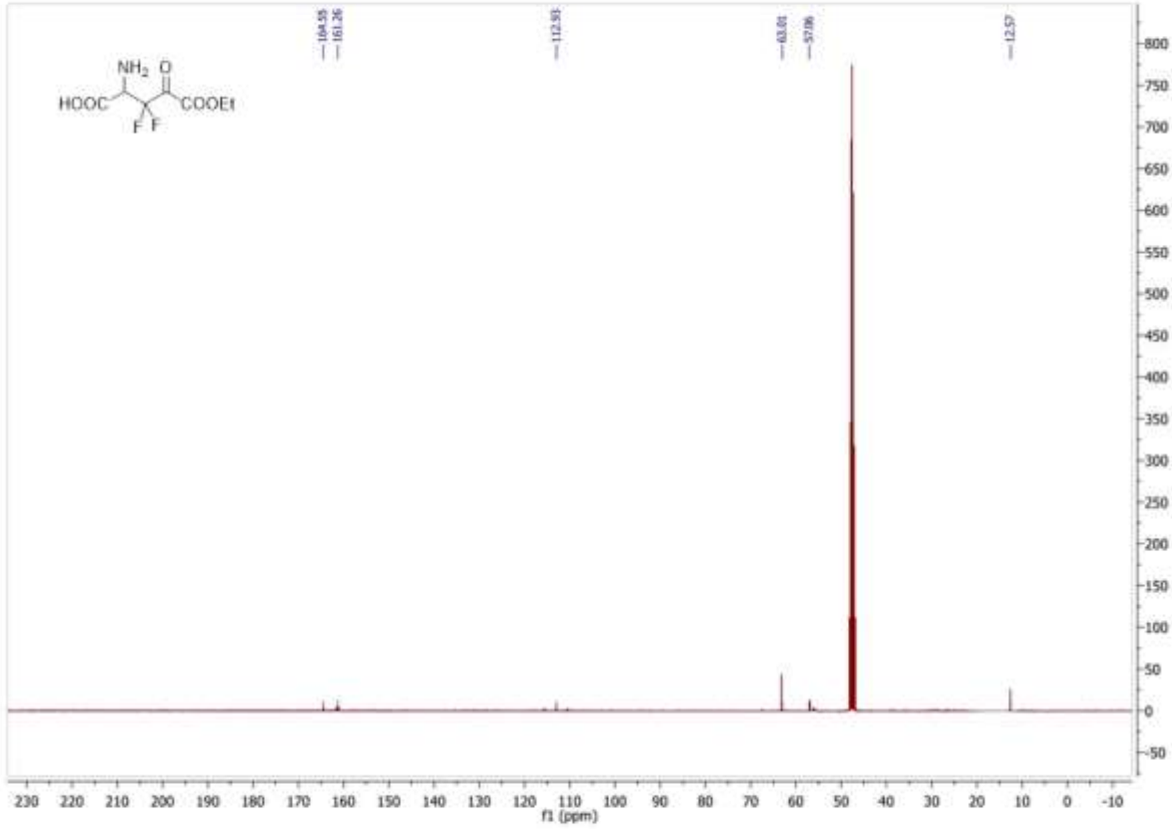

Supplement: Supplemental Material [file IENZ_A_1504040_SM6430.pdf]
